# Elevated microRNA-187 causes cardiac endothelial dysplasia to promote congenital heart disease through inhibition of NIPBL

**DOI:** 10.1172/JCI178355

**Published:** 2024-11-25

**Authors:** Chao Li, Zizheng Tan, Hongdou Li, Xiaoying Yao, Chuyue Peng, Yue Qi, Bo Wu, Tongjin Zhao, Chentao Li, Jianfeng Shen, Hongyan Wang

**Affiliations:** 1 Obstetrics and Gynecology Hospital, State Key Laboratory of Genetic Engineering, Children’s Hospital, and; 2 Shanghai Key Laboratory of Metabolic Remodeling and Health, Institute of Metabolism and Integrative Biology, Fudan University, Shanghai, China.; 3 Prenatal Diagnosis Center of Shenzhen Maternity and Child Healthcare Hospital, Shenzhen, China.; 4 Shanghai Medical College, Fudan University, Shanghai, China.; 5 Department of Ophthalmology, Ninth People’s Hospital, Shanghai JiaoTong University School of Medicine, Shanghai, China.

**Keywords:** Cardiology, Development, Cardiovascular disease, Noncoding RNAs

## Abstract

Cardiac endothelial cells are essential for heart development, and disruption of this process can lead to congenital heart disease (CHD). However, how microRNAs influence cardiac endothelial cells in CHD remains unclear. This study identified elevated microRNA-187 (miR-187) expression in embryonic heart endothelial cells from CHD fetuses. Using a conditional knockin model, we showed that increased miR-187 levels in embryonic endothelial cells induce CHD in homozygous fetal mice, closely mirroring human CHD. Mechanistically, miR-187 targets *NIPBL*, which is responsible for recruiting the cohesin complex and facilitating chromatin accessibility. Consequently, the endothelial cell–specific upregulation of miR-187 inhibited NIPBL, leading to reduced chromatin accessibility and impaired gene expression, which hindered endothelial cell development and ultimately caused heart septal defects and reduced heart size both in vitro and in vivo. Importantly, exogenous miR-187 expression in human cardiac organoids mimicked developmental defects in the cardiac endothelial cells, and this was reversible by NIPBL replenishment. Our findings establish the miR-187/NIPBL axis as a potent regulator that inhibits cardiac endothelial cell development by attenuating the transcription of numerous endothelial genes, with our mouse and human cardiac organoid models effectively replicating severe defects from minor perturbations. This discovery suggests that targeting the miR-187/NIPBL pathway could offer a promising therapeutic approach for CHD.

## Abstract 2

MiR-187 upregulation in fetal cardiac endothelial cells induces CHD, mirroring human cases. Mechanistically, miR-187 targets *NIPBL*, impairing chromatin accessibility and gene expression critical for cardiac endothelial cells development. This study unveils the potent regulatory role of the miR-187/NIPBL axis in CHD pathogenesis.

## Graphical abstract

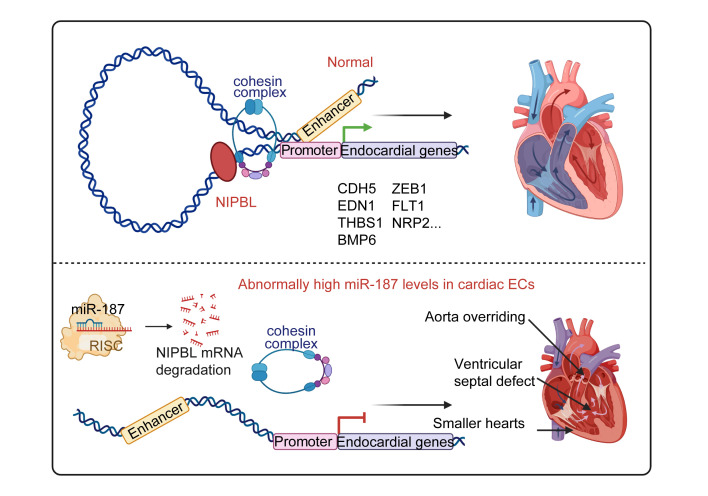

## Introduction

Congenital heart disease (CHD), the most prevalent congenital disorder in newborns (1), includes ventricular septal defects (VSDs) as the predominant form, accounting for approximately 50% of cases. Among VSDs, the perimembranous subtype constitutes approximately 75% (2, 3). Tetralogy of Fallot (TOF), a severe form of CHD that commonly features VSDs (3), serves as a valuable model for studying perimembranous VSDs. Previous research has identified more than 50 gene mutations (4) and de novo copy number variants (5) associated with specific types of CHD. However, over 55% of CHD cases remain unexplained (6). In particular, the genetic factors identified for perimembranous VSDs account for only a small subset of cases (7), leaving a substantial gap in our understanding that warrants further investigation.

In addition to gene mutations, related protein dosage alterations also regulate gene expression. MicroRNAs (miRNAs), short noncoding RNAs, typically interact with the 3′-UTR of target mRNAs, leading to the suppression of protein production and playing a vital role in regulating post-transcriptional gene expression in both physiological and pathological processes of the heart (8). Given their established involvement in various aspects of cardiac development and disease, miRNAs are considered potential pathogenic factors in perimembranous VSDs (9).

During heart septum formation, a specific subset of cardiac endothelial cells located above the future septum undergoes a transformation, giving rise to cardiac cushions. Cardiac endothelial cell dysplasia, defined by impaired differentiation and function, can lead to underdeveloped cardiac cushions, potentially causing congenital heart defects in the valves and atrial septa (10). Several functional miRNAs associated with VSDs have been identified (11), and a few miRNAs in cardiomyocytes have been shown to induce VSDs in transgenic models and cardiomyocyte-specific (12) knockout mouse models (13, 14). In both mouse and human hearts, endothelial cells make up approximately 20%–40% of the cellular composition (15, 16). Genes expressed in endothelial cells, such as *JAG1* (17), play a critical role in heart development, particularly in septal formation. Nevertheless, our understanding of the physiological functions of endothelial cell–specific miRNAs in cardiac septal development is limited.

A recent comprehensive single-cell analysis of cardiogenesis has revealed an intriguing connection between CHD and altered chromatin accessibility specifically in the endothelium (18). Cohesin, a ring-shaped protein complex that attaches to chromosomes, plays a critical role in chromatin accessibility and remodeling, bringing regulatory DNA into close proximity with target DNA (19) and facilitating the folding of the genome into DNA loops (20). Cohesin-mediated loop extrusion and dwell time are essential for determining the positions of replication origins during mitosis (21). During interphase, cohesin contributes to shaping the genome into a 3-dimensional structure and interacts with other regulatory factors to control gene expression (22). In the cohesin loading process, the Nipped-B homolog (NIPBL) is important as a recruiting center (23). Haploinsufficiency of NIPBL due to mutations accounts for approximately 70% of cases of Cornelia de Lange syndrome (CdLS) (24, 25), an inheritable disorder predominantly associated with cardiac septal defects (25). CHD is observed in 14%–70% of individuals with CdLS (26, 27), often involving VSDs and atrial septal defects, accompanied by hypoplastic ventricles (28). Recent studies revealed that 77% of *NIPBL^+/–^* mouse hearts displayed incomplete or completely absent contact between the developing ventricular septum and the cardiac cushion with smaller ventricles than those of wild-type (WT) mice. However, the specific lineage responsible for the increased risk of septal defects remains unclear (29). Therefore, an understanding of the regulatory mechanisms of NIPBL and the role of NIPBL in cardiac endothelial cell development might be crucial.

In this study, we aimed to identify key miRNAs involved in the development of VSDs during cardiac development. For this purpose, we used human cardiac organoids, human embryonic stem cell–derived (hESC-derived) endothelial lineage differentiation, mouse genetic models, and epigenomic and transcriptomic analyses. We investigated and revealed the functional and molecular mechanisms underlying the regulatory role of microRNA-187 (miR-187) in cardiac endothelial cell development. Consequently, our findings demonstrated a critical role for the miR-187/NIPBL signaling pathway in early cardiac endothelial cell differentiation, shedding light on the pathogenic mechanisms underlying CHDs associated with dysregulated miR-187 and attenuated NIPBL expression.

## Results

### Cardiac endothelial cell–specific upregulation of miR-187 is positively associated with the development of TOF.

**Figure 1:**
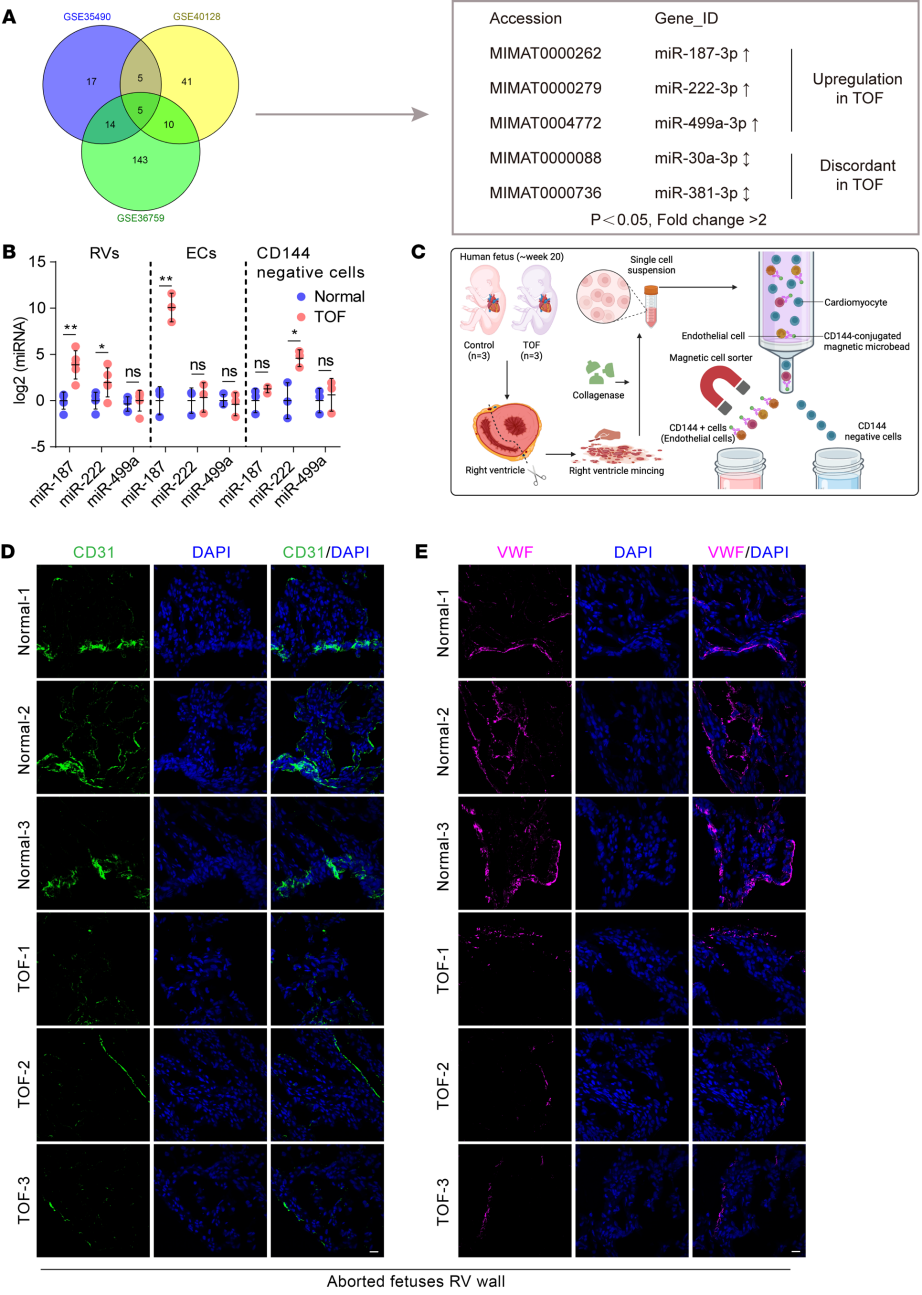
Upregulation of miR-187 expression in the hearts of fetuses with TOF. (**A**) Schematic illustration of the screening process for differentially expressed miRNAs (fold change >2, *P* < 0.05) using 3 independent datasets (GSE35490, GSE36759, and GSE40128). (**B**) RT-qPCR analysis of miR-187, miR-222, and miR-499a levels in the right ventricles (RVs), endothelial cells (ECs), and cardiomyocytes (CMs) of aborted fetuses with TOF (for RVs, *n* = 5; for ECs and CMs, *n* = 3) and normal controls (for RVs, *n* = 5; for ECs and CMs, *n* = 3). U6 was used as an internal control. (**C**) Schematic diagram of the isolation of endothelial cells from human hearts. (**D** and **E**) Representative immunofluorescence staining of endothelial marker CD31 (green, **D**) and VWF (magenta, **E**) in heart sections from right ventricle wall tissues from fetuses aborted at about 20 weeks with TOF (*n* = 3) and normal controls (*n* = 3). DAPI was used for nuclear staining (blue). Scale bars: 20 μm. Data are shown as means **±** SD. ns, *P* > 0.05; *P < 0.05, **P < 0.01. Significance was determined by 1-way ANOVA (**B**).

The nonrestrictive perimembranous VSD was reported to be strongly associated with TOF (3). To identify miRNA involvement in perimembranous development, we performed data mining to identify differentially expressed miRNAs in the right ventricular outflow tract of patients with TOF from 3 datasets (Gene Expression Omnibus GSE35490, GSE40128, and GSE36759). Three upregulated miRNAs (miR-187-3p, miR-222-3p, miR-499a-3p) and 2 discordant miRNAs, miR-30a-3p (up in GSE35490/GSE36759, down in GSE40128) and miR-381-3p (up in GSE36759/GSE40128, down in GSE35490), were identified in the overlapping TOF datasets (Figure 1A). We measured the expression levels of the 3 upregulated miRNAs using right ventricle tissues from aborted fetuses with TOF (Supplemental Table 1; *n* = 3 pairs). Only miR-187 (miR-187-3p, abbreviated as miR-187) and miR-222 showed significantly higher expression in the TOF cases than in the controls (Figure 1B, left – RV lane).

Furthermore, cardiac endothelial cells from the collected right ventricles were isolated using CD144 (VE-cadherin) MicroBeads and magnetic-activated cell sorting (MACS) (Figure 1C). MiR-187 was the only miRNA that was intensely upregulated in endothelial cells marked by CD144 positivity but remained stable in CD144-negative cells (Figure 1B, right [CD144^–^ cells lane]).Meanwhile, except for CD144-positive cells, miR-187 expression also showed no difference in other examined tissues, including the brain, kidney, lung, and liver, between fetuses with TOF and controls (Supplemental Figure 1A; supplemental material available online with this article; https://doi.org/10.1172/JCI178355DS1). Notably, the levels of the most common endothelial markers, CD31 and VWF, were dramatically lower in right ventricle wall tissues from the fetuses with TOF than in the normal controls (Figure 1, D and E). Together, these results suggested that both increased miR-187 levels in endothelial cells and reduced endothelial cells might be involved in CHD onset.

### MiR-187 inhibits the development of normal cardiac endothelial cells.

**Figure 2:**
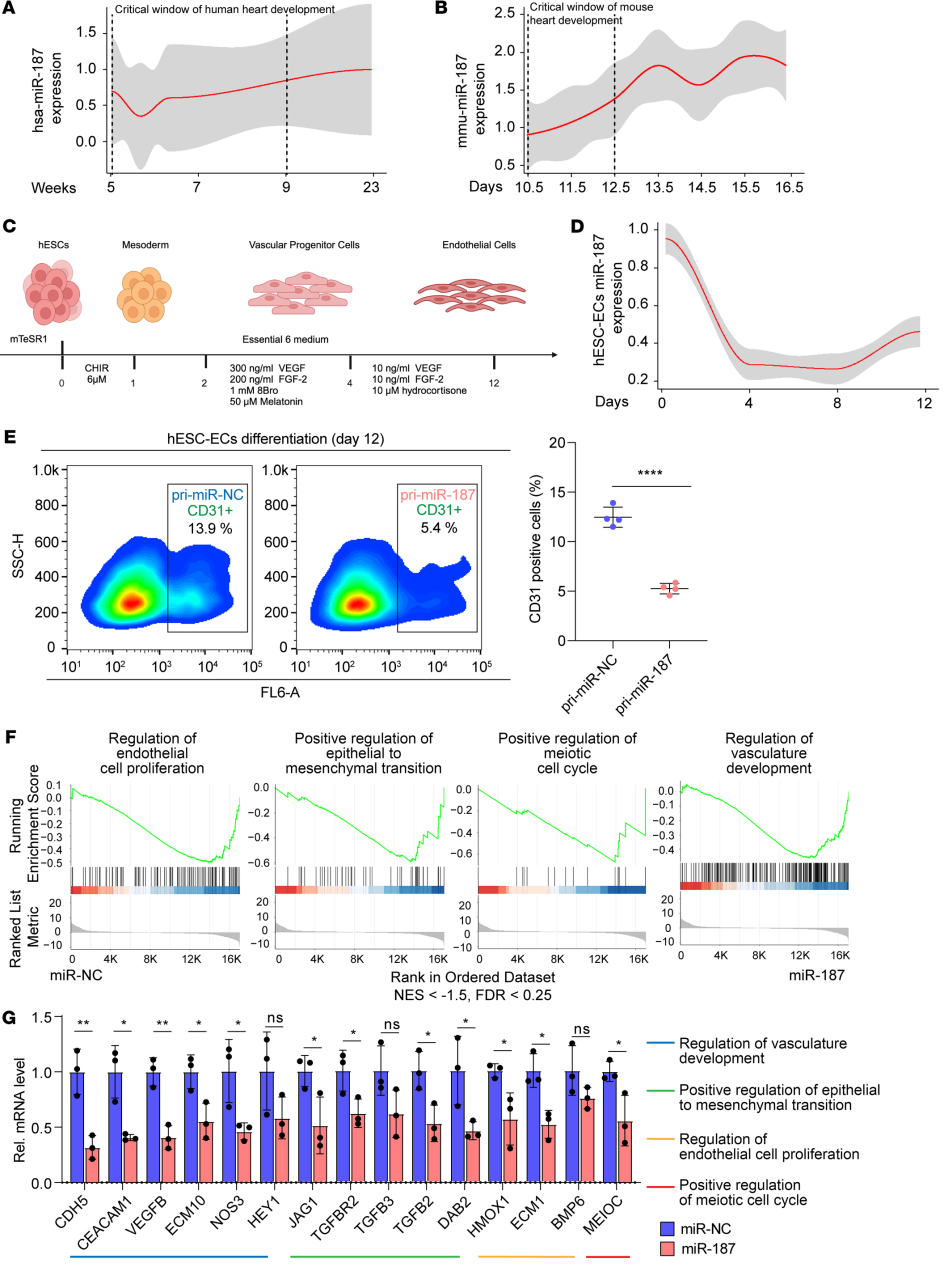
High miR-187 expression levels impair endothelial development. (**A**, **B**, and **D**) Temporal analysis of miR-187 expression during normal human heart development by microarray analyses (**A**) and mouse heart development using the GSE105834, GSE82960, GSE105910, GSE82604, GSE82822, GSE82942, and GSE101175 datasets (**B**) and differentiation of hESCs into endothelial cells by RT-qPCR (*n* = 4) (**D**). (**C**) Schematic of protocol for differentiation from hESCs to endothelial cells. (**E**) FACS analysis and quantification of CD31-positive cells in hESC-EC infection with pri-miR-187 or scramble control by lentivirus (*n* = 4). (**F**) Representative GSEA results for regulation of endothelial cell proliferation (GO:0001936), positive regulation of epithelial-to-mesenchymal transition (GO:0010718), positive regulation of meiotic cell cycle (GO:0051446), and regulation of vasculature development (GO:1901342) gene sets. (**G**) RT-qPCR verification of representative genes of GO:0001936, GO:0010718, GO:0051446, and GO:1901342 gene sets (*n* = 3). U6 or GAPDH was used as an internal control. Data are shown as means ± SD. ns, *P* > 0.05; *P < 0.05, **P < 0.01, ****P < 0.0001. Significance was determined by 1-way ANOVA (**G**) and 2-tailed t test (**E**).

We examined miR-187 levels during normal embryonic development in vivo and investigated how highly expressed miR-187 impaired endothelial cell differentiation in vitro. Compared with the later stages of heart development (week 23), the expression level of miR-187 remained relatively low from week 5 to week 9, which is a critical window for human embryonic heart development, and then gradually increased based on the results of microarray analysis (Figure 2A and Supplemental Figure 1B). Similarly, compared with E15.5, mouse miR-187 was low during the critical window of heart development (E10.5 to E12.5) and subsequently increased in later stages of heart development (Figure 2B and Supplemental Figure 1C). Additionally, a modified approach (Figure 2C) was used to test the expression of those TOF-related miRNAs in differentiation of human induced pluripotent stem cell–derived endothelial cells (30). Unlike the initial expression and subsequent increase of miR-187 in human and mouse hearts, miR-187 expression gradually decreased during endothelial cell differentiation as determined by quantitative reverse transcriptase PCR (RT-qPCR) analysis (Figure 2D and Supplemental Figure 1D). These findings suggest that maintenance of low miR-187 expression during critical stages of heart development is essential for proper early embryonic cardiac endothelial cell development. Predictably, abnormally high miR-187 levels might disrupt cardiac endothelial cell development in the embryonic heart.

To investigate the effect of high levels of miR-187 on endothelial cell function, we constructed an hESC line with stable overexpression of exogenous miR-187 and verified the pluripotency of hESC (Supplemental Figure 2, A and B). Flow cytometry results indicated that the number of endothelial cells (CD31 positive) was significantly decreased on day 12 during hESC-derived endothelial cell (hESC-EC) differentiation in cells with stable miR-187 overexpression (Figure 2E).

Furthermore, gene set enrichment analysis (GSEA) in a human endothelial cell line (EA.hy926) revealed that the regulation of endothelial cell proliferation, epithelial-to-mesenchymal transition (EMT), and meiotic cell cycle vasculature development (FDR < 0.25) were impaired in endothelial cells transfected with miR-187 compared with controls (Figure 2F and Supplemental Figure 2, C–G). RT-qPCR verified that in EA.hy926 cells treated with exogenous miR-187, the pathway markers for endothelial cell proliferation, positive regulation of EMT, positive regulation of the meiotic cell cycle, and regulation of vasculature development were downregulated (Figure 2G). These findings suggest that abnormally high miR-187 expression levels might contribute to endothelial pathogenesis through impaired proliferation and differentiation in an early stage of heart development.

### Endothelial cell–specific miR-187–knockin model mouse recapitulates the phenotype of human CHD.

**Figure 3:**
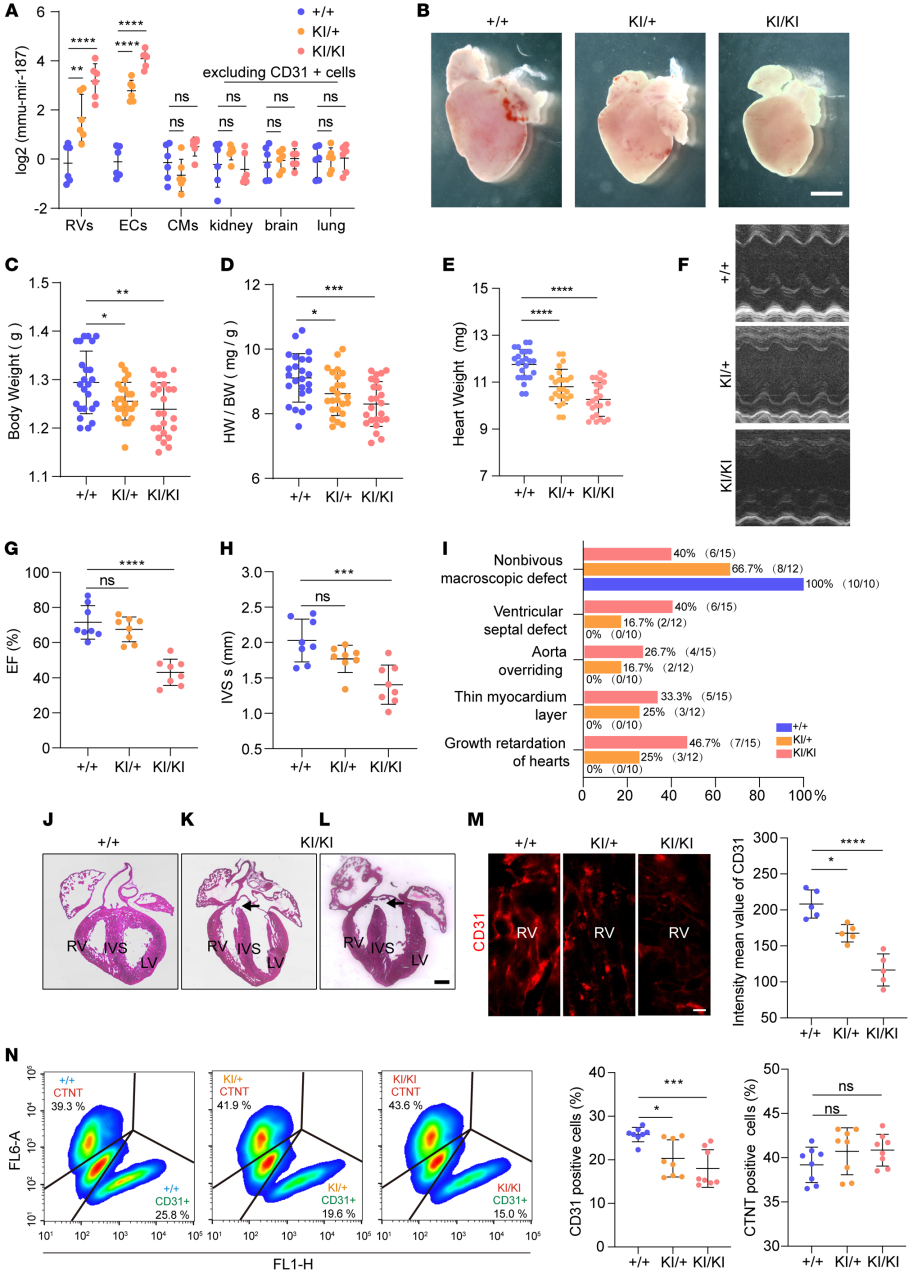
Endothelial cell–specific expression of exogenous miR-187 drives CHD. (**A**) RT-qPCR analysis of mmu-miR-187 levels in the hearts, endothelial cells, cardiomyocytes, and other tissues excluding CD31-positive cells (kidneys, brains, and lungs) of P0 neonatal mice with the indicated genotypes (*n* = 6). (**B**) Stereoscopic images of whole hearts from homozygous, heterozygous miR-187–KI, and control mice at P0. (**C**–**E**) Body weight (**C**), heart weight/body weight ratio (**D**), and heart weight (**E**) of P0.5 neonatal homozygous miR-187–KI and control mice (*n* = 24). (**F**–**H**) Echocardiographic assessment of representative M-mode images of the left ventricle (**F**), ejection fraction (EF) (**G**), and systolic intraventricular septum (IVS s) (**H**) in control mice and miR-187–KI mice (*n* = 8). (**I**) Quantification of cardiac defect number according to stereoscopic images and H&E-stained sections of whole hearts of control mice and homozygous miR-187–KI mice. (**J**–**L**) H&E-stained heart sections from homozygous miR-187–KI and control mice, displaying human-CHD-like phenotypes; for example, the control heart shows a normal septum (**J**), and a miR-187–KI littermate of the animal in **K** and **L** shows VSD (**K**, arrow) and aorta overriding (**L**, arrow) at P0.5. (**M**) Quantification of the intensity mean value of CD31 (red) per field of view (*n* = 5). (**N**) FACS analysis and quantification of CTNT and CD31-positive cells from homozygous, heterozygous miR-187–KI, and control mice at P0.5 (*n* = 8). Scale bars: 1,000 μm (**B**), 200 μm (**J**–**L**), 5 μm (**M**). Data are shown as means ± SD. ns, *P* > 0.05; *P < 0.05, **P < 0.01, ***P < 0.001, ****P < 0.0001. Significance was determined by 1-way ANOVA (**A**, **C**–**E**, **G**, **H**, **M**, and **N**) and Pearson’s χ^2^ test (**I**).

MiR-187–knockin (KI) mice were conditionally generated using the *Tek* (*Tie2*) promoter to limit the expression of exogenous miR-187 specifically in endothelial cells (Supplemental Figure 3A). MiR-187 expression levels in the right ventricles of homozygous KI mice were approximately 10-fold higher than those in controls (Figure 3A), similar to increased miR-187 expression in TOF patients compared with controls. The upregulated cardiac miR-187 was exclusively limited to the cardiac endothelial cells between KI/KI and KI/+ mice (Figure 3A), showing no significant changes in the compared cardiomyocytes (Figure 3A). The embryonic lethality in the homozygous miR-187–KI mice on day 10 was revealed by the interbreeding Mendelian ratio of 14:60:33 in KI/KI, KI/+, and +/+ offspring (Supplemental Figure 3B). Anatomical analysis revealed that the hearts of homozygous and heterozygous miR-187–KI pups at P0 were smaller than those of WT pups (Figure 3B), although the size of the cardiomyocytes did not change (Supplemental Figure 3C). The body weight of the miR-187–KI pups was significantly lower than that of the control pups (Figure 3C and Supplemental Figure 3, D–F). Remarkably, the heart/body weight ratio and heart weight dramatically decreased in KI/KI mice compared with WT mice (Figure 3, D and E, and Supplemental Table 2). These results indicate that the overexpression of endothelial cell–derived miR-187 prominently reduced heart weight in addition to reducing whole-body weight in KI/KI pups.

The ejection fraction and intraventricular septum evaluated by echocardiography in miR-187–KI mice were significantly lower than those in WT mice (Figure 3, F–H). We examined the cardiac phenotype using H&E-stained sections at P0.5 and found VSDs displayed in 6 of 15 miR-187–KI/KI mice (Figure 3, I–K) and 2 of 12 KI/+ mice (Supplemental Figure 3G); aorta overriding in 4 of 15 KI/KI mice (Figure 3L); thin myocardium layer in 5 of 15 KI/KI mice and 3 of 12 KI/+ mice (Figure 3H and Supplemental Figure 3, H–N); and smaller hearts in 7 of 15 KI/KI mice and 3 of 12 KI/+ mice (Figure 3B). The number of cardiac endothelial cells undergoing mitosis indicated by the marker pH3 was also decreased in KI/KI and KI/+ mice (Supplemental Figure 3O). Consistent with the in vitro results (Figure 2E), the expression of CD31, a marker of endothelial cells, was also significantly reduced in the right ventricle of miR-187–KI mice compared with controls (Figure 3M). FACS analyzed the number of cardiomyocytes and endothelial cells in the heart tissues from P0.5 mice. The FACS analysis results showed that the proportion of endothelial cells in the heart tissues of miR-187–KI mice was significantly lower than that in WT controls, but the proportion of cardiomyocytes between miR-187–KI mice and WT control mice showed no significant difference (Figure 3N). Both the reduction in mitotic cardiac endothelial cells and decreased endothelial cell numbers exhibited in KI mice are similar to what was observed in CHD patients (18). These results demonstrate that embryonic endothelial cell–specific expression of exogenous miR-187 in mice could recapitulate the phenotypes of human CHD.

### Doxorubicin-induced upregulation of miR-187 inhibits the growth of human heart-forming organoids.

Being a risk factor for CHD, doxorubicin can significantly induce the expression levels of miR-187 in the cardiomyocytes (31–33). Embryonic stem cell–induced human heart-forming organoids (HFOs) serve as a valuable in vitro model that can mimic CHD phenotypes caused by genetic and environmental factors (34, 35). HFOs can simulate the early stages of cardiac development in vitro and have various cell types, including endothelial cells (34). Therefore, we hypothesized that doxorubicin-treated HFOs would exhibit increased miR-187 expression and display heart defects like those in miR-187–KI mice. We engineered 3-dimensional HFOs through biphasic regulation of the WNT signaling pathway (34) (Supplemental Figure 4A), comprising a myocardial layer surrounded by an inner core of cardiac endothelial cells and encased by proepicardial outer layer anlagen (Supplemental Figure 4, B–F). In doxorubicin-treated HFOs, miR-187 expression was significantly elevated in comparison with controls (Supplemental Figure 5A). The addition of a miR-187 inhibitor restored miR-187 levels to normal (Supplemental Figure 5A). Doxorubicin treatment inhibited the growth of HFOs, as evidenced by significantly reduced volume and area in comparison with the control group; however, supplementation with miR-187 inhibitor restored HFO growth (Supplemental Figure 5, B–D). Through immunofluorescence detection of pH3-positive cells, doxorubicin-treated HFOs demonstrated markedly diminished mitotic capability compared with the control group (Supplemental Figure 5E). We found that doxorubicin treatment inhibited endothelial cell differentiation in CD31-labeled day 10 HFOs (Supplemental Figure 5F). Supplementation with a miR-187 inhibitor restored the mitotic capability of HFOs and enhanced the differentiation of endothelial cells (Supplemental Figure 5, E and F). These results indicate that elevated miR-187 expression in HFOs can pathologically simulate the cardiac phenotype observed in miR-187–KI mice (Figure 3, B and M).

### NIPBL is a target of miR-187 during cardiac endothelial cell development.

**Figure 4:**
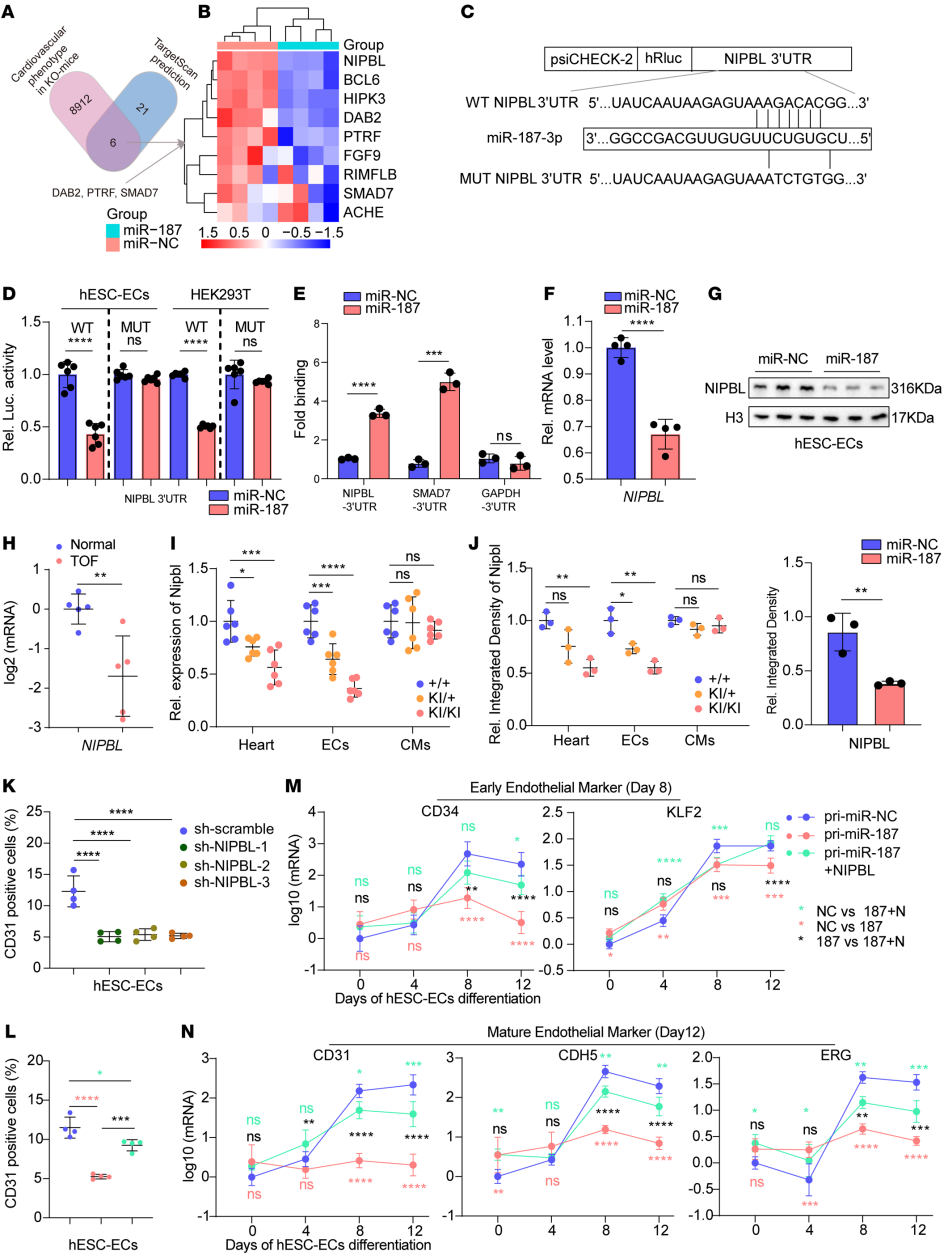
MiR-187 targets NIPBL and disturbs endothelial development. (**A**) Schematic illustration of the screening approach for target genes of miR-187 using TargetScan prediction, MGI database, and RT-qPCR verification. (**B**) Cluster analysis of RT-qPCR results showing expression levels of candidate target genes of miR-187 in hESC-ECs transfected with miR-187 mimic. (**C**) Schematic illustration of luciferase reporters containing WT and mutant miR-187 binding sites in the NIPBL 3′-UTR. (**D**) Luciferase assays of hESC-ECs or HEK293T cells cotransfected with miR-187 or scramble control and luciferase reporter plasmids containing WT or mutant NIPBL 3′-UTR. (**E**) The human NIPBL 3′-UTR pulled down by biotin–miR-187 or biotin-scramble control was quantified by RT-qPCR in hESC-ECs. SMAD7–3′-UTR and GAPDH–3′-UTR serve as positive and negative controls, respectively. (**F** and **G**) RT-qPCR and Western blotting was used to analyze mRNA (**F**) and protein (**G**, WB) levels of NIPBL in hESC-ECs transfected with miR-187 or scrambled control, with grayscale analysis used to quantify the NIPBL protein (**G**, statistical analysis). (**H**) RT-qPCR analysis of mRNA levels of NIPBL in RVs of aborted fetuses with TOF and control fetuses (*n* = 5). (**I** and **J**) mRNA (**I**) and protein (**J**) level of NIPBL in whole hearts, cardiac endothelial cells, and cardiomyocytes of P0 neonatal mice of the indicated genotypes (*n* = 6). (**K** and **L**) FACS quantification of CD31-positive cells in hESC-EC infection with sh-NIPBL-1, -2, -3, sh-scramble (**K**), pri-miR-187, pri-miR-187+NIPBL, or scramble control by lentivirus (*n* = 4) (**L**). (**M** and **N**) RT-qPCR analyses of expression levels of various markers for early endothelial (**M**) and mature endothelial cells (**N**) during differentiation from hESCs to endothelial cells (*n* = 4). GAPDH or H3 was used as an internal control. Data are shown as means **±** SD. ns, *P* > 0.05; *P < 0.05, **P < 0.01, ***P < 0.001, ****P < 0.0001. Significance was determined by 1-way ANOVA (**D**, **E**, and **I**–**L**), 2-way ANOVA (**M** and **N**), and 2-tailed *t* test (**F**–**H**).

To search for miR-187 targets involved in the pathogenesis of CHD, we performed a bioinformatics analysis of the TargetScan database (https://www.targetscan.org/vert_71/) and identified 21 genes containing conserved miR-187 target sites (Supplemental Table 3). In the Mouse Genome Informatics (MGI) mouse phenotypic database, 6 of 21 possible target genes showed CHD-related phenotypes in individual knockout mice. Together with the 3 previously reported miR-187 target genes, *DAB2* (36), *PTRF* (37), and *SMAD7* (38) (Figure 4A), the RT-qPCR results verified 6 downregulated genes and 3 upregulated genes among a total of 9 target genes (Figure 4B). *NIPBL* was the most significantly downregulated gene, which plays crucial roles in developing septal defects and functions coordinately in cohesin loading on chromatin and transcription signaling (39), so we finally selected *NIPBL* for further investigation. Bioinformatics analysis showed that miR-187 is conserved across multiple species, and the binding sequence of miR-187 in the 3′-UTR of *NIPBL* is also conserved between mice and humans (Supplemental Figure 6, A and B). We hypothesized that the upregulation of miR-187 expression overinhibited *NIPBL* expression to impair proper gene expression, ultimately contributing to CHD occurrence.

First, the luciferase reporter assays indicated that miR-187 bound directly to the 3′-UTR of *NIPBL* (Figure 4, C and D). We also demonstrated through RNA immunoprecipitation experiments that miR-187 bound to the 3′-UTR of *NIPBL*, with *SMAD7* being used as a positive control and *GAPDH* as a negative control (Figure 4E). Then, the expression of endogenous NIPBL could be reduced in vitro by the expression of exogenous miR-187 at both the mRNA (Figure 4F) and protein levels (Figure 4G, Western blot [WB]) as quantified by grayscale analysis (Figure 4G, statistical analysis) in hESC-ECs. Moreover, RT-qPCR showed that *NIPBL* mRNA levels were significantly lower in the right ventricle of human fetuses with TOF than in control fetuses (Figure 4H). Meanwhile, such differences were not found in other parallelly compared tissues (Supplemental Figure 6C). Exclusively, both the in vivo *NIPBL* mRNA and protein levels were significantly lower in heart endothelial cells from miR-187–KI mice than in controls (Figure 4, I and J, and Supplemental Figure 6D). These results demonstrate that miR-187 directly targets and negatively regulates *NIPBL*.

### The miR-187/NIPBL axis is critical for maintaining endothelial differentiation.

We created lentivirus-mediated knockdown of NIPBL expression in human stem cells (Supplemental Figure 6E) to test whether the upregulation of miR-187/NIPBL or reduced NIPBL impairs endothelial differentiation from hESC-ECs. FACS results showed that the percentage of endothelial cells indicated by the CD31 marker markedly decreased with stable knockdown of NIPBL expression in hESC-ECs compared with that of the scramble control group (Figure 4K and Supplemental Figure 6F). We overexpressed both miR-187 and NIPBL in embryonic stem cells and evaluated pluripotency (Supplemental Figure 6, G and H). Consistently, the FACS results also showed that the percentages of CD31-positive cells in miR-187–hESC-ECs were significantly lower than those in miR-187/NIPBL–hESC-ECs, which coexpressed both miR-187 and NIPBL (Figure 4L and Supplemental Figure 6I). Overexpression of NIPBL was able to reverse the expression reduction of many key endothelial genes independently induced by exogenous miR-187 (Figure 4, M and N, and Supplemental Figure 7, A–D). Moreover, inhibition of miR-187 expression by a miR-187–specific inhibitor resulted in enhanced differentiation efficiency and mitotic capacity of hESC-ECs compared with the control group (Supplemental Figure 8, A–D). These results strengthen the role of the miR-187/NIPBL axis in regulating endothelial cell development and mitosis.

To determine whether miR-187 mediates endothelial cell differentiation and mitosis in cardiac development via other target genes such as *SMAD7*, a member of the TGF-β signaling pathway (38), we coexpressed SMAD7 into miR-187–overexpressing hESC-ECs. The results showed that SMAD7 could partially reverse the inhibition of endothelial cell differentiation and mitosis induced by miR-187 (Supplemental Figure 8, C and D), which was weaker than the recovery efficacy produced by overexpressed NIPBL (Supplemental Figure 8, C and D). This suggests that *NIPBL* may be the main effector target of miR-187 in regulating endothelial cell development. Meanwhile, both arterial endothelial cell development genes, including *GJA5*, *HAND2*, and *ANXA1*, and venous endothelial genes, including *NR2F2*, did not change in the mature stage of endothelial cells overexpressing miR-187 (Supplemental Figure 8, E and F), which coincided with the phenotype of no apparent defects in vascular development observed in miR-187–KI mice. Collectively, the above results demonstrate that the upregulated miR-187 disturbs cardiac endothelial cell differentiation by inhibiting the expression of NIPBL.

### The miR-187/NIPBL axis inhibits endothelial cell migration and tube formation.

As suggested by the RNA-Seq results that miR-187 repressed core gene expression for migration, EMT, and angiogenesis in endothelial cells (Figure 2F and Supplemental Figure 9), we speculated that the functions related to these genes might be regulated by the miR-187/NIPBL axis. The results of wound healing assays indicated that the closure areas of the miR-187 groups were significantly smaller than those of the miR-187/NIPBL–coexpressing controls, and conversely, reduction of miR-187 expression enhanced hESC-EC migration (Supplemental Figure 10A). WB analysis showed that miR-187–hESC-ECs had lower N-cadherin levels compared with miR-NC–hESC-ECs (noncompetitive miRNAs), indicating impaired mesenchymal differentiation, which could be reversed by NIPBL overexpression or by miR-187 inhibitors (Supplemental Figure 10, B and C). Overexpression of miR-187 reduced the mRNA levels of the EMT signaling markers CD31 and CDH5 in mature endothelial cells (Figure 4N). During heart development, endothelial cells generate mesenchymal cells with migratory and plastic properties via EMT, the primary source of coronary vascular endothelial cells (40). Indeed, defects in angiogenesis and downregulated expression of angiogenesis-associated genes were found with miR-187 overexpression (Figure 2F). MiR-187–transfected hESC-ECs displayed shorter tube length, reduced junction number, and impaired mesh area in the tube formation assay, which could be partially rescued by NIPBL supplementation or miR-187 inhibitors (Supplemental Figure 10D). The above results emphasized NIPBL as a key target gene of miR-187 in regulation of endothelial cell function. The findings that the miR-187/NIPBL axis inhibits endothelial cell migration and angiogenesis in vitro would help us interpret the etiology of overexpression of miR-187 in endothelial cells in vivo.

### Overexpression of NIPBL reverses the small-heart phenotype induced by miR-187 in human HFOs.

The use of human embryonic stem cells to induce differentiation of HFOs can avoid species differences and enable rapid gene editing (35). Therefore, we chose an HFO model to test whether NIPBL supplementation can reverse the miR-187–induced phenotype. HFOs stably expressing miR-187, miR-187/NIPBL, or scramble hESCs (Supplemental Figure 2, A and B, and Supplemental Figure 6, G and H) were used to study the effects of miR-187 on cardiac endothelial cell development.

**Figure 5:**
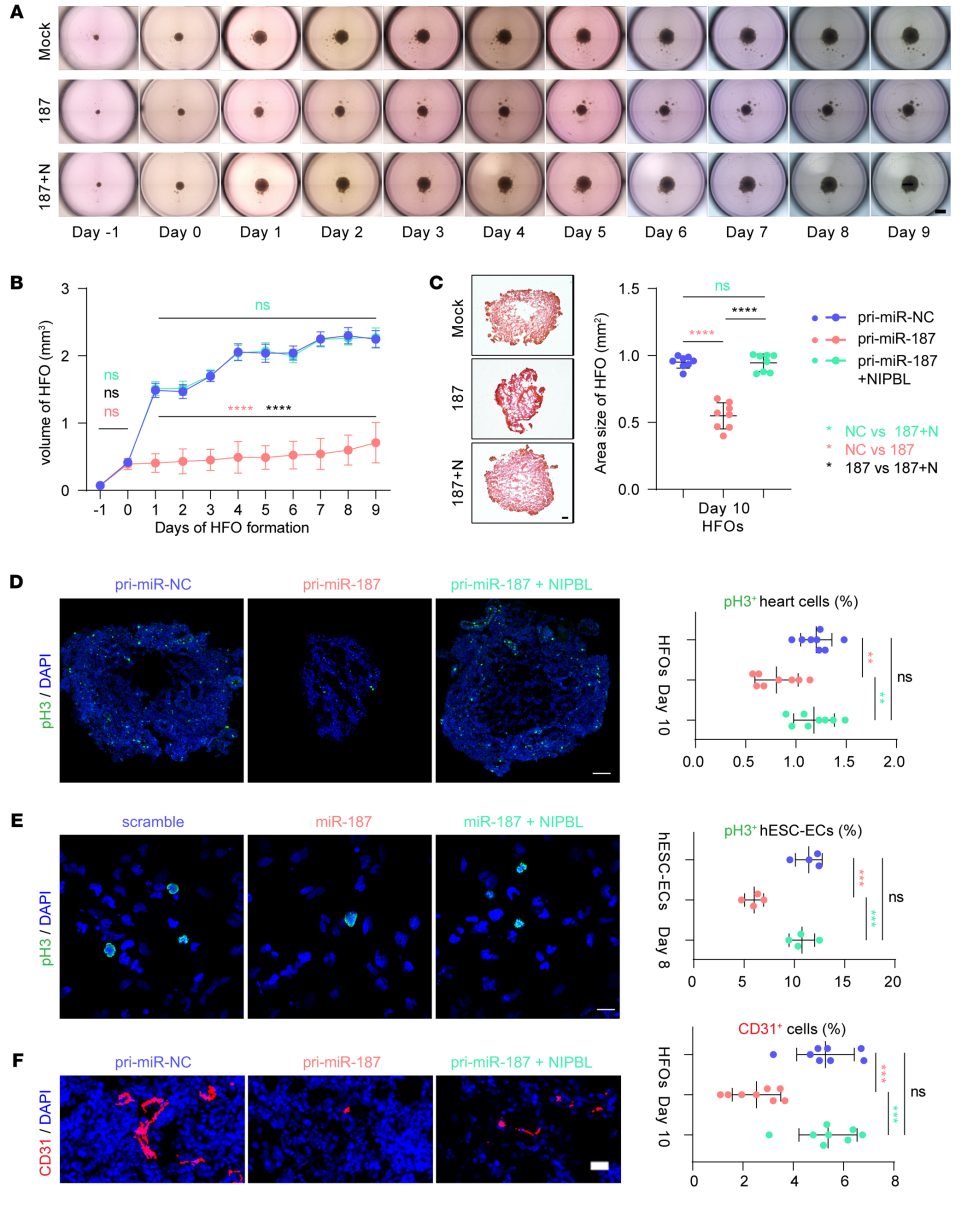
NIPBL rescues delayed HFO formation induced by miR-187. (**A**) The development of HFOs from day −1 until day 9 of mock, miR-187, and miR-187/NIPBL differentiation. (**B**) Quantification of volume of HFOs (*n* = 32) from day −1 until day 9 of mock, miR-187, and miR-187/NIPBL differentiation. (**C**) H&E staining (left) and quantification (*n* = 8) (right) of area for mock, miR-187, and miR-187/NIPBL HFOs at day 10. (**D**) Immunostainings for pH3 (green, left) and quantification (*n* = 8) (right) show the number of mitotic mock, miR-187, and miR-187/NIPBL HFO cells at day 10. (**E**) Immunostainings for pH3 (green, left) and quantification (*n* = 4) (right) show the number of mitotic mock, miR-187, and miR-187/NIPBL hESC-ECs at day 8. (**F**) Representative immunofluorescence staining (red, left) and quantification (*n* = 8) (right) of the endothelial cell marker CD31 in mock, miR-187, and miR-187/NIPBL HFO cells at day 10. DAPI was used for nuclear staining (blue). Scale bars: 1 mm (**A**), 100 μm (**C**), 20 μm (**F**). Data are shown as means ± SD. ns, *P* > 0.05; **P < 0.01, ***P < 0.001, ****P < 0.0001. Significance was determined by 1-way ANOVA (**C**–**F**) and 2-way ANOVA (**B**).

Morphologically, miR-187–HFOs grew and dilated significantly more slowly between days 1 and 9 compared with the standard rate of growth and expansion maintained by the scramble-HFOs (Figure 5, A and B, and Supplemental Figure 11, A–L). NIPBL supplementation in miR-187–hESCs on differentiation day 1 could recover the standard rate of growth and expansion (Figure 5, A and B, and Supplemental Figure 11, A–L). H&E staining showed that the area of miR-187–HFOs was smaller than that of both miR-187/NIPBL–HFOs and scramble-HFOs, while the latter two showed no significant change (Figure 5C).

Immunofluorescence results focused on the mitotic marker pH3 showed that expression of miR-187 significantly suppressed the mitosis of HFOs, while supplementation with NIPBL restored such suppressed proliferation (Figure 5D). Specifically, immunofluorescence shows that miR-187 inhibited the proliferation of endothelial cells, which was rescued by the expression of NIPBL (Figure 5E). The immunofluorescence results indicated that the proportion of CD31-positive cells in miR-187–HFOs was substantially lower than that in miR-187/NIPBL–HFOs and in scramble-HFOs and the percentage of CD31-positive cells in the latter two did not change significantly (Figure 5F). These data identify the pathogenic effect induced by overexpression of miR-187 through attenuation of NIPBL.

### MiR-187 affects chromatin accessibility and gene expression in cardiac endothelial cells.

**Figure 6:**
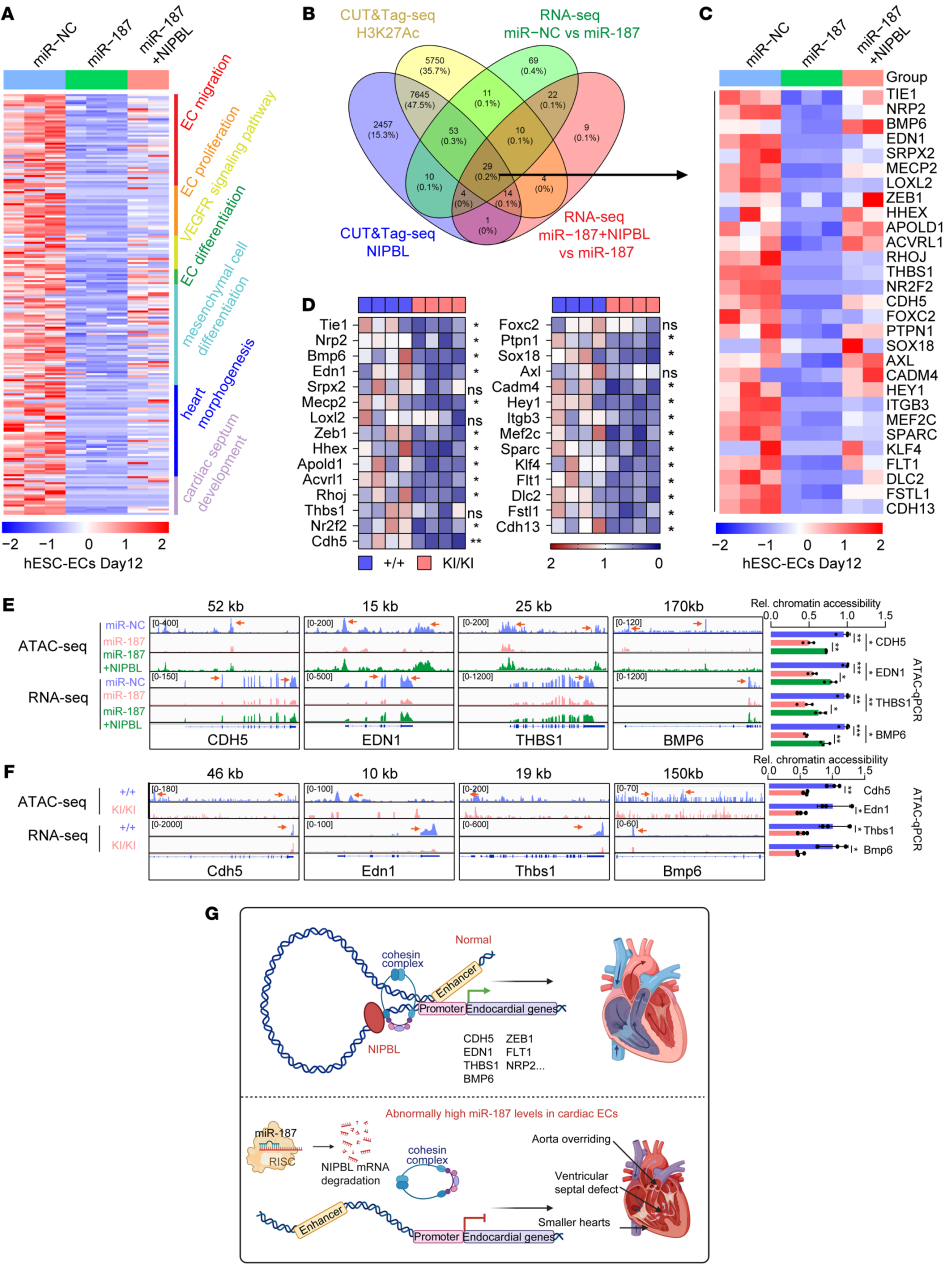
MiR-187 reduces endocardial gene expression and chromatin accessibility and inhibits endothelial cell migration and tube formation. (**A**) Heatmap of RNA-Seq analyses of expression levels of 208 core genes represented in Gene Ontology (GO) terms of GSEA, involved in endothelial cell migration, proliferation, differentiation, vascular endothelial growth factor (VEGF) signaling pathway, mesenchymal cell differentiation, heart morphogenesis, and cardiac septum development for miR-NC–hESC-ECs, miR-187–hESC-ECs, and miR-187/NIPBL–hESC-ECs. (**B**) Schematic illustration of the screening approach for downstream genes of miR-187/NIPBL axis using NIPBL, H3K27Ac CUT&TAG-seq and miR-NC–hESC-EC, miR-187–hESC-EC, and miR-187/NIPBL–hESC-EC RNA-Seq. (**C**) Heatmap of RNA-Seq analyses of 29 screened genes for miR-NC–hESC-ECs, miR-187–hESC-ECs, and miR-187/NIPBL–hESC-ECs. (**D**) RT-qPCR analysis of 29 screened genes in endocardial cells of WT and miR-187–KI mice (*n* = 4). GAPDH was used as an internal control. (**E** and **F**) Genome Browser displays representative views of ATAC-seq and RNA-Seq signals for the indicated genes (left), while ATAC-qPCR quantification shows the chromatin accessibility of these genes (*n* = 4) (right), comparing hESC-ECs with miR-NC, miR-187, miR-187+NIPBL (**E**), as well as mice with +/+ and KI/KI genotypes (**F**). (**G**) Schematic diagram of the role of the miR-187/*NIPBL* axis in the pathogenesis of CHD. Data are shown as means **±** SD. ns, *P* > 0.05; *P < 0.05, **P < 0.01, ***P < 0.001. Significance was determined by 1-way ANOVA (**D**–**F**).

RNA-Seq results showed that compared with those of scramble cells or miR-187/NIPBL–hESC-ECs, downregulated genes in miR-187–hESC-ECs were represented in Gene Ontology (GO) terms of GSEA (FDR < 0.25) and involved in endothelial cell migration, proliferation, differentiation, vascular endothelial growth factor (VEGF) signaling pathway, mesenchymal cell differentiation, heart morphogenesis, and cardiac septum development (Figure 6A and Supplemental Figure 9). We identified a total of 208 target genes downregulated by upregulation of miR-187 that were responsible for restricting cardiac endothelial cell growth (Figure 6A), of which 65 downregulated genes could be restored by NIPBL supplementation (Supplemental Figure 12, A–C). To explore whether these genes are subject to NIPBL-mediated transcriptional regulation, we performed CUT&Tag sequencing (CUT&Tag-seq) of NIPBL compared with that of H3K27Ac as control for the active promoter and enhancer regions in hESC-ECs (Supplemental Figure 13, A–C). We detected widespread binding to the promoter and predominantly active enhancer regions (Supplemental Figure 13, A and B). For comparison, we included genome-wide CUT&Tag-seq data for H3K27Ac in hESC-ECs, showing its preferential binding (Supplemental Figure 13, A–C). After narrowing down the results from the shared binding region genes between NIPBL and H3K27Ac, we identified 29 differentially expressed cardiac endothelial cell development–associated genes that were transcriptionally regulated by the miR-187/NIPBL axis (Figure 6, B and C). Consistently, RT-qPCR results found that most of the 29 genes were downregulated in the cardiac endothelial cells of miR-187–KI mice compared with WT mice (Figure 6D).

To investigate the chromatin accessibility changes of genes regulated by the miR-187/NIPBL axis, we conducted assay for transposase-accessible chromatin with sequencing (ATAC-seq) on hESC-ECs overexpressing miR-187 or co-overexpressing miR-187/NIPBL. Some critical genes showed decreased ATAC-seq peaks and RNA-Seq peaks on miR-187–overexpressing hESC-ECs, which were restored upon supplementation with NIPBL (Figure 6E, left). This result was further confirmed by ATAC-qPCR (Figure 6E, right) and RNA-qPCR (Supplemental Figure 13D). The RNA-Seq results showed that the gene expression levels positively correlated with the distribution of the ATAC-seq signal (Supplemental Figure 13E). To verify the in vitro results, we plotted the chromatin accessibility in CD31 magnetic bead–labeled endothelial cells from the heart tissues of P0 mice. The overall accessibility across the genome was reduced in the cardiac endothelial cells of miR-187–KI mice (Supplemental Figure 14A), and the top peaks that lost accessibility were enriched in the promoter region (Supplemental Figure 14B). Predictably, the miR-187/NIPBL axis affected genome-wide chromatin accessibility, and the actively transcribed development-related genes in cardiac endothelial cells were significantly impacted. The epigenomic landscape of cardiac endothelial cells in miR-187–KI mice was further compared with that in WT mice to reveal the miR-187/*NIPBL* axis regulation model in vivo. ATAC-seq results showed that the accessibility of some critical genes among the 29 genes was decreased in the miR-187–KI group compared with that in the control mice (Figure 6F). All the above findings prove that overexpression of miR-187 reduces the accessibility of chromatin, inhibits cardiac endothelial cell gene transcription, and provides molecular support for the observed phenotype of cardiac endothelial cell malformation (Figure 6G). Moreover, our zebrafish model suggests a critical role for miR-187 in CHD (Supplemental Figure 15). Overall, these findings highlight miR-187’s crucial role in disrupting chromatin accessibility and gene expression in cardiac endothelial cells, contributing to CHD.

## Discussion

MiR-187 plays essential roles in the control of cancerous cell proliferation, osteoblast (39, 40) and keratinocyte differentiation, and regulation of the immune response and insulin metabolism (41, 42). Here, we report a CHD mouse model created by cardiac endothelial cell–specific overexpression of miR-187. Usually, the promoters *myh6* and *myh7* are used to drive cardiomyocyte-specific gene expression or deletion for CHD studies (43, 44), so CHD caused by endothelial cell abnormalities might be grossly underestimated, although nearly one-third of heart cells are endothelial either in humans or in mice (15, 17). Hence, our miR-187–KI mice provide a pioneer mouse model to elucidate miR-187–mediated regulation of cardiac endothelial cells and heart development.

NIPBL is needed to adequately load cohesin onto chromosomes, loop extrusion on chromosomal loops, and close target genes with distant regulatory factors to activate gene transcription (25, 45). The enrichment of *NIPBL* mutations was detected in patients with atrioventricular septal defect (46), and an inadequate dose of NIPBL resulted in defective heart development in mice (29). In this study, we identified miR-187 as highly expressed in cardiac endothelial cells from TOF patients and showed miR-187 as a master regulator of NIPBL downregulation at the post-transcriptional level. Consequently, being the first identified regulator beyond *NIPBL* mutations, overexpressed miR-187 decreases the accessibility of endothelial development–related genes adjacent to chromatin by targeting *NIPBL*, which finally leads to incomplete endothelial development, septal defects, and smaller hearts.

Mutations in *NIPBL* are the most common cause of CdLS, with 60%–70% of patients characterized by abnormal cardiac development, alongside anxiety-related behaviors and other malformations (47). *NIPBL* binding sites are enriched within the dysregulated gene’s promoter region, and these genes’ expression is significantly reduced in CdLS-predisposed individuals because of *NIPBL* mutations. Our study suggests that overexpression of miR-187 produces pathological effects on cardiac development by targeting *NIPBL*, which could be restored by *NIPBL* supplementation. Even though miR-187 targets multiple genes, such as *SMAD7*, it was found that *NIPBL* might be the primary downstream effector of miR-187 specifically in endothelial cells. Our result also confirms that the cardiac developmental abnormalities caused by *NIPBL* dosage deficiency are mainly caused by excessive inhibition of genes related to cardiac endothelial development. Considering the mechanism of how *NIPBL* mutations in CdLS cause CHD, we provide a possible explanation that the shared dysregulation of endothelial development could be attributable to *NIPBL* mutations or miR-187–induced NIPBL downregulation. Additionally, no differences were observed in the open field test for miR-187–KI mice (Supplemental Figure 16, A and B). In the light/dark transition test, although the frequency of transitions between the light and dark compartments remained unchanged (Supplemental Figure 16C), miR-187–KI mice spent significantly more time in the dark compartment, indicating heightened anxiety-like behavior (Supplemental Figure 16D). This suggests that miR-187–KI mice partially exhibit the anxiety phenotype seen in CdLS.

Given the human genomic background, the present HFOs employed in our study worked as a good in vitro model to recapitulate smaller hearts for studying developmental mechanisms, function, and pathogenesis in a dish, providing insight into the nature of CHD and offering an ideal opportunity for potential high-throughput drug discovery for adult cardiopathy. We applied HFOs to demonstrate that miR-187–overexpressing HFOs exhibited slow growth reminiscent of the cardiac malformations observed in *NIPBL*-knockout mice (29). Subsequently, supplementation with NIPBL restored normal cardiac morphology both in miR-187–HFOs and in our miR-187–KI mice. These results suggest that targeting miR-187/NIPBL could be a promising therapeutic strategy for CHD. Additionally, the miR-187–mediated dysregulation of endothelial development resulting in CHD could be partially attributable to the inhibition of *NIPBL*. Our research sheds light on the role of miR-187/NIPBL signaling in controlling endothelial and cardiac development as potential therapeutic targets for the prevention of CHD.

### Limitation.

Although we found that doxorubicin can induce an increase in miR-187 expression in HFOs in vitro, showing a phenotype like that of miR-187–KI mice, the in vivo factors that trigger the upregulation of miR-187 in CHD cardiac endothelial cells have not yet been determined. Notably, circulating miR-187 in adults with hypertension-induced heart failure is highly expressed (48), indicating the dual function of miR-187 in both the developing heart and the functional adult heart.

## Methods

### Sex as a biological variable.

Our study examined male and female animals, and similar findings are reported for both sexes.

### Human tissue samples.

Our subjects were fetuses miscarried at about 20 weeks with nonsyndromic TOF (i.e., no 22q11.2 deletion; *n* = 5), and sex- and age-matched miscarried fetuses without TOF (*n* = 5) were used as controls. The diagnosis was obtained by echocardiography and confirmed during an anomaly scan. Written informed consent was obtained from a parent or legal guardian after they reviewed the consent document and had their questions answered. The right ventricle, brain, liver, lung, and kidney tissues were surgically excised from miscarried fetuses with TOF or control. All experiments in this study were conducted with approval from the Medical Ethics Committee at the Obstetrics and Gynecology Hospital of Fudan University.

### Endothelial cell MACS separation.

Single-cell suspensions of human and mouse hearts were prepared through tissue mincing and enzymatic digestion using an isolation enzyme kit (Thermo Fisher Scientific, 88281). Human and mouse hearts were collected and minced into approximately 1 mm blocks. Minced hearts were digested in 200 μL Isolation Enzyme 1 and Isolation Enzyme 2 (Thermo Fisher Scientific, 88281) in HBSS (Thermo Fisher Scientific, 88281) at 37°C for 30 minutes with tissue suspension triturated every 10 minutes. Five hundred microliters cold buffer consisting of 0.5% FBS (Corning, CGR-35-081-CV) and 2 mM EDTA (Invitrogen, AM9260G) in PBS (Gibco, 10010049) was added to stop digestion, and the resulting cell suspensions were filtered through a 40 μm cell strainer (Falcon, 352340) before centrifugation at 300*g* for 5 minutes at 4°C. Cell numbers were determined, and cell pellets were resuspended in 80 μL of buffer per 10^7^ total cells. Twenty microliters of CD144 (VE-Cadherin) MicroBeads, human (Miltenyi Biotec, 130-097-857), or CD31 MicroBeads, mouse (Miltenyi Biotec, 130-097-418), were added for human hearts or mouse hearts, respectively. Mix well and incubate for 15 minutes at 4°C. Cells were washed by addition of 1 mL buffer and centrifuged at 300*g* for 5 minutes at 4°C. For detection of positive cell rate, the cell pellet was resuspended in 500 μL buffer, and human CD31-APC (eBioscience, 17-0319-42) or mouse CD31-APC (eBioscience, 17-0311-82) staining antibody was added before incubation for 15 minutes in the dark at 4°C. LS Columns were placed in a MACS separator (Miltenyi Biotec, 130-042-303) and rinsed 3 times with 1 mL of buffer. The cell suspension was added to the column and washed 3 times with 1 mL of buffer before the magnetically labeled cells were flushed out by firm pushing of the plunger into the column.

### Cardiomyocyte separation.

Collagenase II was used to dissociate mouse cardiac tissue at 37°C for 1 hour, followed by filtration through a 100 μm mesh to collect single-cell suspension. The suspension was treated with 1 mL of red blood cell lysis buffer at room temperature for 1 hour, centrifuged at 300*g* for 5 minutes to remove the supernatant, and subsequently incubated with 647 Mouse Anti–Cardiac Troponin T (BD Pharmingen, 565744) in PBS at 4°C for 30 minutes in the dark. Cardiac Troponin T–labeled cardiomyocytes were collected using the BD FACSAria cell sorter (BD Biosciences) and used for subsequent experiments.

### Real-time RT-qPCR and RNA-Seq.

Cells or tissue samples were extracted using TRIzol and isolated with a miRNeasy Mini Kit (QIAGEN, 217004) following the manufacturer’s recommendations. For miRNA detection, 2 μg of total RNA was used to synthesize cDNA using a miRNA First-Strand cDNA Synthesis Kit (GeneCopoeia, QP014). RT-qPCRs were next performed in 96-well plates using a miRNA RT-qPCR Detection Kit (GeneCopoeia, QP016). For mRNA detection, 500 ng of total RNA was used to synthesize cDNA using a HiScript III 1st Strand cDNA Synthesis Kit (+gDNA wiper) (Vazyme, R312). The mRNA levels were determined by RT-qPCR using HiScript III All-in-one RT SuperMix Perfect for qPCR (Vazyme, R333). All RT-qPCRs were performed using the Applied Biosystems QuantStudio 1 Real-Time PCR System in a volume of 20 μL. Data were quantified using the comparative Ct method, with *U6* or *GAPDH* as a reference gene. Relative gene expression levels were calculated using the 2^−ΔΔCt^ method. A list of the qPCR primers used in this study can be found in Supplemental Table 4.

Human endothelial cells (EA.hy926), hESC-ECs, and cardiac endothelial cells from P0 neonatal KI/KI and WT control mice were collected for RNA-Seq assay performed by BGI Genomics and APExBIO, respectively.

### ATAC-seq and ATAC-qPCR.

To prepare the sample for ATAC-qPCR, 50,000 viable cells were pelleted at 500 relative centrifugal force (RCF) at 4°C for 5 minutes, and the supernatant was aspirated. Next, 50 μL of cold ATAC–Resuspension Buffer (RSB) containing 0.1% NP-40, 0.1% Tween-20, and 0.01% digitonin was added to the cell pellet and pipetted up and down 3 times. The mixture was then incubated on ice for 3 minutes, and the lysis was washed out with 1 mL of cold ATAC-RSB containing 0.1% Tween-20 but no NP-40 or digitonin. The nuclei were pelleted at 500 RCF for 10 minutes at 4°C, and the supernatant was aspirated. The cell pellet was then resuspended in 50 μL of transposition mixture (25 μL 2× TD buffer (Vazyme, TD711-01), 2.5 μL transposase [100 nM final], 16.5 μL PBS, 0.5 μL 1% digitonin, 0.5 μL 10% Tween-20, 5 μL H_2_O) by pipetting up and down 6 times. The reaction was incubated at 37°C for 30 minutes. The DNA was subsequently purified with VAHTS DNA Clean Beads (Vazyme, N411-01) and amplified with barcode primers using the TruePrep DNA Library Prep Kit (Vazyme, TD501-01). Subsequent sequencing and data analysis were outsourced to APExBIO in Shanghai, China. ATAC-qPCR was performed using the same library construction method as in ATAC-seq. The ATAC libraries were subsequently adapted for RT-qPCR using specific primers designed based on previous articles (49).

### CUT&Tag.

The CUT&Tag assay used the Hyperactive Universal CUT&Tag Assay Kit for Illumina (Vazyme, TD903). In brief, 10^5^ endothelial cells were collected and washed with 500 μL of wash buffer. The cells were then bound to ConA beads for 10 minutes at 25°C. Subsequently, the cells were incubated with 1 μg of *NIPBL* (Bethyl Laboratories, A301-779A-T) or H3K27Ac (Abcam, ab177178) antibody at 4°C overnight. The next day, anti-rabbit IgG was added and incubated for 1 hour at 25°C. After 3 washes with DIG wash buffer (Vazyme, HD-102), the cells were incubated with 0.04 μM pA/G-transposon (pA/G-Tnp) for 1 hour at 25°C. After 3 washes with DIG 300 buffer, the cells were resuspended in tagmentation buffer and incubated at 37°C for 1 hour. Tagmentation was stopped by addition of proteinase K, buffer LB (Vazyme, HD-102), and DNA extract beads. The cells were then incubated at 55°C for 10 minutes, and the unbound liquid was removed after plating of the cells on a magnet. The beads were gently rinsed twice with 80% ethanol, and the DNA was eluted with double-distilled water. Libraries were constructed using the TruePrep Index Kit V2 for Illumina (Vazyme, TD202). Subsequent sequencing and data analysis were outsourced to GENEWIZ Biotechnology Co. Ltd.

### Cell culture.

Human embryonic stem cell line H9 (WA09, obtained from WiCell Research Institute) was cultured on Matrigel Matrix–precoated (1:200; Corning, 354277) 6-well plates at 10 μg/cm^2^ growth area in mTeSR1 Plus medium (StemCell Technologies, 100-0276) in a humidified incubator at 37°C with 5% CO_2_. The cells were seeded at a density of 5 × 10^5^ cells per well, and the medium was replaced every 2 days.

EA.hy926 and HEK293T cells were obtained from the ATCC and cultured in DMEM (Gibco, 11995073) with 10% FBS (Corning, 35076111) and 1‰ Plasmocin (InvivoGen, ant-mpt) at 37°C and 5% CO_2_.

hESC-ECs were cultured in gelatin-coated (Sigma-Aldrich, G2500-100G) 6-well plates in EGM-2 Endothelial Cell Growth Medium-2 Bullet Kit (Lonza, CC-3162).

### Differentiation of hESC-derived endothelial cells.

The protocol to generate endothelial cells from hESCs was modified from the previously reported method (30). Briefly, hESCs were seeded on Matrigel-coated plates in mTeSR1 Plus medium to 30% confluence. At 30% confluence, the hESCs were pushed toward the mesodermal lineage by treatment with 6 μM CHIR-99021 (Selleck, S1263) in Essential 6 (E6; Gibco, A1516401) medium for 1 day, followed by a non-treatment in E6 medium for 1 day. At day 2 of differentiation, the cells were subjected to a differentiation medium composed of E6 medium supplemented with 300 ng/mL Recombinant Human VEGF (R&D Systems, 293-VE-010/CF), 200 ng/mL Recombinant Human FGF-2 (PeproTech 100-18B), 1 mM 8-bromoadenosine 3′,5′-cyclic monophosphate sodium salt monohydrate (8Bro; Sigma-Aldrich, 858463-25MG), and 50 μM melatonin (Sigma-Aldrich, M5250-1G) for 48 hours. From day 4 to day 12, the culture medium was changed every 48 hours into E6 medium supplemented with 10 ng/mL VEGF, 10 ng/mL bFGF, and 10 μM hydrocortisone (Selleck, S1696).

### Lentivirus infection.

To produce hESC-ECs expressing pri-miR-187 and/or *NIPBL*, human embryonic kidney (HEK) 293T cells were grown to 80% confluence on 100 mm plates. Cotransfection of 12 μg of pLJM1-pri-miR-187 and/or PCDH-*NIPBL* with the packaging plasmids (7.8 μg of pMDL, 6 μg of pREV, and 4.2 μg of pVSVG from Addgene) was carried out using Lipofectamine 2000 (Invitrogen, 11668019). After 48 hours, the viral supernatant was collected, concentrated using PEG 8000, and stored at –80°C. hESCs were infected with the pri-miR-187– and/or *NIPBL*-expressing lentivirus and then selected with puromycin (0.2 μg/mL; InvivoGen, ant-pr-1) for 2 weeks. RT-qPCR was performed to confirm the expression of miR-187 and *NIPBL*. The resulting hESC-ECs were pri-miR-187– and/or *NIPBL*-expressing hESC-ECs.

### Immunofluorescence staining.

Immunofluorescence staining was performed on hESC-ECs plated on Matrigel-coated glass-bottom dishes (NEST, Wuxi, China; 801001) or heart/HFO sections using a procedure previously described below in *Formation and culture of HFOs*. The cells or heart sections were fixed and permeabilized with 0.5% Triton X-100 (Sangon, A110694-0100) in PBS for 5 minutes and then blocked with 5% BSA (Sangon, A600332-0100) in PBS for 1 hour at room temperature. The samples were incubated with primary antibodies diluted in 3% BSA blocking solution overnight at 4°C. The samples were then incubated with a secondary antibody and stained with DAPI. The slides were observed under a confocal microscope (Carl Zeiss, LSM880).

### Flow cytometry.

The pri-miR-187, pri-miR-187/NIPBL, and scramble hESC-ECs were treated with StemPro Accutase Cell Dissociation Reagent (Gibco, A1110501) and incubated with APC-conjugated Mouse Anti–Human CD31 (WM59) (Thermo Fisher Scientific, 17-0319-42) in PBS at 4°C for 30 minutes in the dark. Collagenase II was used to dissociate mouse cardiac tissue at 37°C for 1 hour, followed by filtration through a 100 μm mesh to collect single-cell suspension. The suspension was treated with 1 mL of red blood cell lysis buffer at room temperature for 1 hour, centrifuged at 300*g* for 5 minutes to remove the supernatant, and subsequently incubated with FITC-conjugated Rat Anti–Mouse CD31 (WM59) (BD Pharmingen, 553372) and 647 Mouse Anti–Cardiac Troponin T (BD Pharmingen, 565744) in PBS at 4°C for 30 minutes in the dark. The cells were then sorted using a BD FACSCalibur (BD Biosciences) or Gallios (Beckman Coulter) flow cytometer. The resulting data were analyzed using FlowJo (BD Biosciences).

### Mouse studies.

The experimental procedures followed the Administrative Panel on Laboratory Animal Care protocol and the institutional guidelines by the Medical Ethics Committee at the Obstetrics and Gynecology Hospital of Fudan University. Rosa26 site-specific miR-187–knockin mice, also known as miR-187–KI mice, were created using the CRISPR/Cas9 system on a C57BL/6J background. These mice expressed a single copy of exogenous mmu-miR-187, controlled by the mouse *Tek* (*Tie2*) promoter.

To prepare for microinjection, capped mRNAs for Cas9 were generated using the mMESSAGE mMACHINE in vitro transcription kit (Invitrogen, AM1344) following the manufacturer’s instructions. The RNA’s integrity was verified by electrophoresis on a 1% agarose gel after denaturation using the loading buffer provided in the Invitrogen kit. Standard plasmid DNA preparation was used, followed by extraction with phenol/chloroform (Sangon, PD0419-1). The DNA was then diluted to 10 ng/μL with sterile microinjection TE buffer (0.1 mM EDTA, 10 mM Tris, pH 7.5; Solarbio, T1140) and stored at −80°C until the injection. We ensured RNase-free DNA by incubating it with in vitro–transcribed RNA at 37°C for 1 hour and analyzing the mix on a 1% agarose gel after denaturation using the loading buffer.

To generate Rosa26 (R26) site-specific miR-187–knockin mice, a donor plasmid containing a mouse miR-187 genomic fragment (5′-TCAGGCTACAACACAGGACCCGGGCGCTGCTCTGACCCCTCGTGTCTTGTGTTGCAGCCGG-3′) and flanking region controlled by a *Tek* promoter, a *Tek* enhancer, and a rabbit globin polyA signal sequence was constructed. The Cas9 mRNA and a single-guide RNA (sgRNA) targeting the R26 locus were generated, and the donor vector, Cas9 mRNA, and sgRNA (5′-GGCAGGCTTAAAGGCTAACC-3′) were co-microinjected into fertilized eggs from C57BL/6J mice, which were then transferred to pseudopregnant mice. The injection mixes contained 5 ng/μL DNA and 50 ng/μL of in vitro–transcribed Cas9 mRNA in microinjection TE buffer. Stable Mendelian transmission was confirmed, and RT-qPCR verified endothelial cell–specific expression of mmu-miR-187. The injection mixes were prepared before each injection by mixing of equal volumes of 10 ng/μL DNA solution and 100 ng/μL mRNA solution.

To confirm the site-specific insertions in the animals, we conducted 3 PCR tests: one for the junction at the 5′ end, one for the junction at the 3′ end, and one located internally within the transgene. The genomic DNA from their offspring was analyzed to confirm positive homologous recombination by PCR. After obtaining the heterozygous miR-187–KI mice, we established homozygous mice by backcrossing them with WT C57BL/6J mice and self-crossing the heterozygous mice. These lines were maintained by breeding of homozygous animals and exhibited normal fertility.

### Echocardiographic studies.

Mice were maintained on a heating platform to keep their body temperature at 36.5°C–37.5°C. The mice were anesthetized with 2% isoflurane and then kept under mild anesthesia during the echocardiographic procedure. Cardiac ultrasound was performed using the Vevo770 imaging system. Initially, the long and short axes of the mouse heart were visualized in B-mode, followed by analysis of the short axis in M-mode.

### Open field test.

Each mouse was placed in a corner of the open field apparatus (40 × 40 × 30 cm) with an illumination level of 100 lux. The number of entries into the central area (20 × 20 cm) and the duration spent there were recorded over a 10-minute period.

### Light/dark transition test.

The light/dark transition test was conducted using a cage (21 × 42 × 25 cm) divided into 2 equal compartments by a partition with a door. One compartment was brightly lit (390 lux), while the other remained dark (2 lux). Mice were placed in the dark compartment and allowed to freely move between the 2 compartments for 10 minutes with the door open. Transition frequency and time spent in each compartment were recorded using ImageLD software (Wayne Rasband, NIH).

### Histological analysis.

The hearts collected at E13.5 or P0 were fixed with 4% paraformaldehyde (pH 7.4; Sigma-Aldrich, P6148-1kg) for 30 or 50 minutes and then embedded in paraffin (Sangon, A606115). They were then sectioned at a thickness of 10 μm and subjected to hematoxylin and eosin (H&E) staining (Sangon, E607318-0200) for routine histological examination using a light microscope.

### Transfection.

The miRNA mimic (B02004) and miRNA inhibitor (B03004) were obtained from GenePharma and used for transfection experiments. To purify endothelial cells, endothelial cells derived from hESCs were purified by MACS at least once, reaching a minimum purity of 90%. Transfection of miRNA mimic, miRNA inhibitor, siRNA, or negative control was carried out using Lipofectamine RNAiMAX (Invitrogen, 13778075), while cotransfection of miR-187 mimic and *NIPBL*-expressing plasmids was performed using Lipofectamine 3000 (Invitrogen, L3000015). Cells were collected 48 hours after transfection.

### Antibodies.

Primary antibodies against the following proteins were used in this study: anti-SOX2 antibody (1:500 for immunofluorescence [IF]; Cell Signaling Technology, 3579T), anti-GAPDH antibody (1:5,000 for WB; Proteintech, 60004-1), anti–human CD31 (1:500 for IF; Abcam, ab9498), anti–mouse CD31 (1:500 for IF; BD Pharmingen, 557355), anti–von Willebrand Factor (1:500 for IF; Abcam, ab6994), FITC-conjugated mouse anti–human CD31 (WM59) (1:50 for FACS; BD Pharmingen, 557508), anti–human CD31 (PECAM-1) monoclonal antibody, APC (1:50 for FACS; eBioscience, 17-0311-82), wheat germ agglutinin, Alexa Fluor 488 conjugate (1:500 for IF; Invitrogen, W11261), anti-NIPBL antibody (1:1,000 for WB; 1:50 for CUT&Tag, Bethyl Laboratories, A301-779A-T), anti–histone H3 antibody (1:1,000 for WB; Cell Signaling Technology, 4499), anti–human phospho–histone H3 (Ser10) antibody (1:500 for IF; Cell Signaling Technology, 53348T), anti–mouse phospho–histone H3 antibody (1:500 for IF; Sigma-Aldrich, 06-570), anti–α-actinin antibody (1:500 for IF; Sigma-Aldrich, A7732), anti-WT1 antibody (1:500 for IF; Abcam, ab89901), anti-NFAT2 antibody (1:500 for IF; Abcam, ab25916), anti-H3K27Ac antibody (1:50 for CUT&Tag; Abcam, ab177178), and anti–normal rabbit IgG (1:50 for CUT&Tag; Cell Signaling Technology, 2729).

The secondary antibodies were goat anti-rabbit Alexa Fluor 488 antibody (1:500 for IF; Invitrogen, A-11008), goat anti-mouse Alexa Fluor 594 antibody (1:500 for IF; Invitrogen, A-11005), HRP-conjugated Affinipure Goat Anti-Rabbit Antibody (1:10,000 for WB; Proteintech, SA00001-4), and HRP-conjugated Affinipure Goat Anti-Mouse Antibody (1:10,000 for WB; Proteintech, SA00001-1).

### Plasmids.

The psiCHECK2-*NIPBL*-3′UTR luciferase reporter plasmid was created by amplifying a 449 bp fragment of the *NIPBL* 3′-UTR from human genomic DNA through PCR and cloning it into the XhoI and NotI sites of psiCHECK-2 (Promega). To generate the mutations plasmid corresponding to miR-187 binding sites, the plasmid of psiCHECK2-*NIPBL*-3′UTR-MUT was subjected to site-directed mutagenesis through PCR, and the resulting mutations were verified by DNA sequencing.

For the lentiviral vector pLJM1-pri-miR-187, a 586 bp human genomic DNA fragment, including pri-miR-187, was amplified by PCR and cloned into the NheI and EcoRI sites of pLJM1 (Addgene).

The *NIPBL* expression plasmids were constructed by cloning of the cDNA of *NIPBL* into pCDH-4HA.

### Immunoblot analysis.

In WB analysis, cells were washed with cold PBS and then lysed in cold Western lysis buffer (Beyotime, p0013) with a protease inhibitor cocktail (Roche, 04693132001). A standard procedure was used for the immunoblot analysis of total protein from the whole-cell lysate. *GAPDH* or *H3* was used as an internal control to normalize the protein loading.

### Luciferase reporter assay.

The luciferase reporter plasmid psiCHECK2-*NIPBL*-3′-UTR or mutants were cotransfected into HEK293T or EA.hy926 cells seeded in 24-well plates along with 100 nM miR-187 mimic or miR-NC mimic and Lipofectamine 3000. After 36 hours, the cells were washed 3 times with cold PBS and lysed in a passive lysis buffer. Luciferase activity was measured using a Dual-Luciferase Assay System (Promega, E1960) on a GloMax-Multi Detection System plate reader (Promega).

### miRNA pull-down assay.

In the miRNA pull-down experiment, biotin-labeled double-stranded miR-187 mimic or miR-NC mimic was transfected into hESC-ECs with Lipofectamine RNAiMAX Transfection Reagent. After 24 hours, the cells were harvested, and RNA binding protein (RNP) complexes with the target mRNAs were pulled down by Dynabeads M-280 Streptavidin (Invitrogen, 11205D). To determine the binding specificity of miR-187 to *NIPBL* mRNA, RT-qPCR analyzed the target mRNAs, and the enrichment of the target mRNAs was calculated as follows: (*NIPBL* mRNA pulled down by miR-187 / *NIPBL* mRNA pulled down by miR-NC mimic) / (biotin–miR-187 input / biotin–miR-NC mimic input). The experiments were performed at least 3 times, with 3 replicates for each set.

### Formation and culture of HFOs.

The protocol of HFO formation was modified from previous publications (34). The hESCs were maintained on Matrigel Matrix (1:200; Corning, 354277) in mTeSR1 Plus medium. For HFO formation, hESCs were detached, and 3 × 10^4^ cells per well were seeded in a U-shaped ultra-low-attachment 96-well plate (NEST, 701101) in mTeSR1 Plus medium. The plate was incubated to allow one aggregate per well to form overnight. On day 0, each aggregate was embedded in a Matrigel (Corning, 356231) droplet. Differentiation was initiated on day 0 by replacing the medium with RPMI 1640 medium containing B27 supplement without insulin (RB–) and supplemented with 7.5 μM CHIR-99021. After 24 hours, the medium was exchanged by RB−, and on day 1, RB– supplemented with 5 μM IWR-1 (Sigma-Aldrich, I0161) was added for 48 hours and then exchanged by RB− on day 3. From day 7 onward, aggregates were cultivated in RPMI 1640 medium containing B27 supplement with insulin (Gibco, 17504044) (RB+). Differentiation was completed on day 7. HFOs were analyzed between day 8 and day 10, and pictures were taken of the whole HFOs using a Castor X1 high-throughput cell analyzer (Countstar). Doxorubicin (500 nM) was administered during days –1 to 10 of HFOs.

### Migration assay.

The effect of miR-187 and *NIPBL* on hESC-EC migration was assessed using wound healing assays. A total of 1 × 10^5^ cells were seeded in 6-well plates and allowed to culture for 24 hours. After 48 hours, transfection was performed using Lipofectamine 3000 with miR-187 mimic only, coexpressed miR-187/NIPBL, or scramble control. The cells were cultured until they reached confluence. Subsequently, scratches were created on the cell layers using a 1 mL pipette tip. The recovered area of the scratches was evaluated after 24 hours using an inverted light microscope.

### Tube formation assay.

hESC-ECs were seeded at 1 × 10^4^ cells/cm^2^ density on a 24-well plate coated with 250 μL of Matrigel (Corning, 356231) in EGM-2 medium. The plate was then incubated for 24 hours at 37°C in a 5% CO_2_ atmosphere. After incubation, the medium was removed, and the plates were washed with PBS. The formation of capillary-like structures was observed using an inverted light microscope. Tube formation was quantified using ImageJ 1.52a software (Wayne Rasband, NIH) and the Angiogenesis Analyzer plug-in (Gilles Carpentier, Université Paris Est Créteil Val de Marne, Créteil, France).

### Zebrafish studies.

In a zebrafish study, exogenous dre-miR-187-3p mimic or negative control (all at 20 pM) was microinjected into fertilized cmlc2-DsRed (labeling CM nucleus) or cmlc2-EGFP (labeling CM membrane) zebrafish embryos. Zebrafish cardiac morphology was measured with confocal microscopy 72 hours after fertilization.

### Statistics.

Data are presented as mean values with corresponding standard deviations (SDs). Tukey’s multiple-comparison test was used for Figure 3, A, C–H, M, and N, Figure 4, I–N, Figure 5, C–F, Supplemental Figure 3, C, E, F, and L–N, Supplemental Figure 5, A and C–F, Supplemental Figure 6E, Supplemental Figure 8, C–F, Supplemental Figure 10, A, C, and D, and Supplemental Figure 13D. Other statistical significance of the differences between groups was determined using 2-sided Student’s *t* tests, and *P* values are reported. Differences in phenotype frequencies (Supplemental Figure 3B) between the KI/KI, KI/+, and +/+ mice were evaluated using Pearson’s χ^2^ test. The significance level is denoted by asterisks: *P < 0.05, **P < 0.01, ***P < 0.001, and ****P < 0.0001.

### Study approval.

All procedures using mice for the current study were approved by the Institute of Developmental Biology and Molecular Medicine of Fudan University. All procedures using human specimens for the current study were approved by the Institutional Review Board of Fudan University. All experiments involving human tissue samples were performed following the Declaration of Helsinki. All experiments involving human tissue samples and animals were conducted with approval from the Medical Ethics Committee at the Obstetrics and Gynecology Hospital of Fudan University.

### Data availability.

All data are available upon reasonable request. The datasets generated during this study were uploaded to the Gene Expression Omnibus database under the following accession codes: GSE275849, GSE275950, and GSE275951 for RNA-Seq; GSE276221 and GSE276222 for ATAC-seq; GSE275850 for CUT&Tag-seq. Supporting data values are available in the Supporting Data Values file.

## Author contributions

CL, ZT, JS, and HW designed the study. CL, ZT, HL, CP, and YQ conducted experiments. CL and ZT performed data mining, RNA-Seq profiling, sequencing analysis, and cell and mouse experiments. CL and ZT assisted with HFOs and hESC-EC culture and differentiation systems. CL and XY optimized the tissue dissociation protocol and collected TOF samples. CL, ZT, BW, TJZ, CTL, and HW interpreted the results, and CL, ZT, HL, YQ, JS, and HW wrote the manuscript. HW and JS supervised the project and provided financial support.

## Supplementary Material

Supplemental data

Unedited blot and gel images

Supplemental table 1

Supplemental table 2

Supplemental table 3

Supplemental table 4

Supporting data values

## Version 1 11/25/2024

In-Press Preview

## Version 2 01/02/2025

Electronic publication
