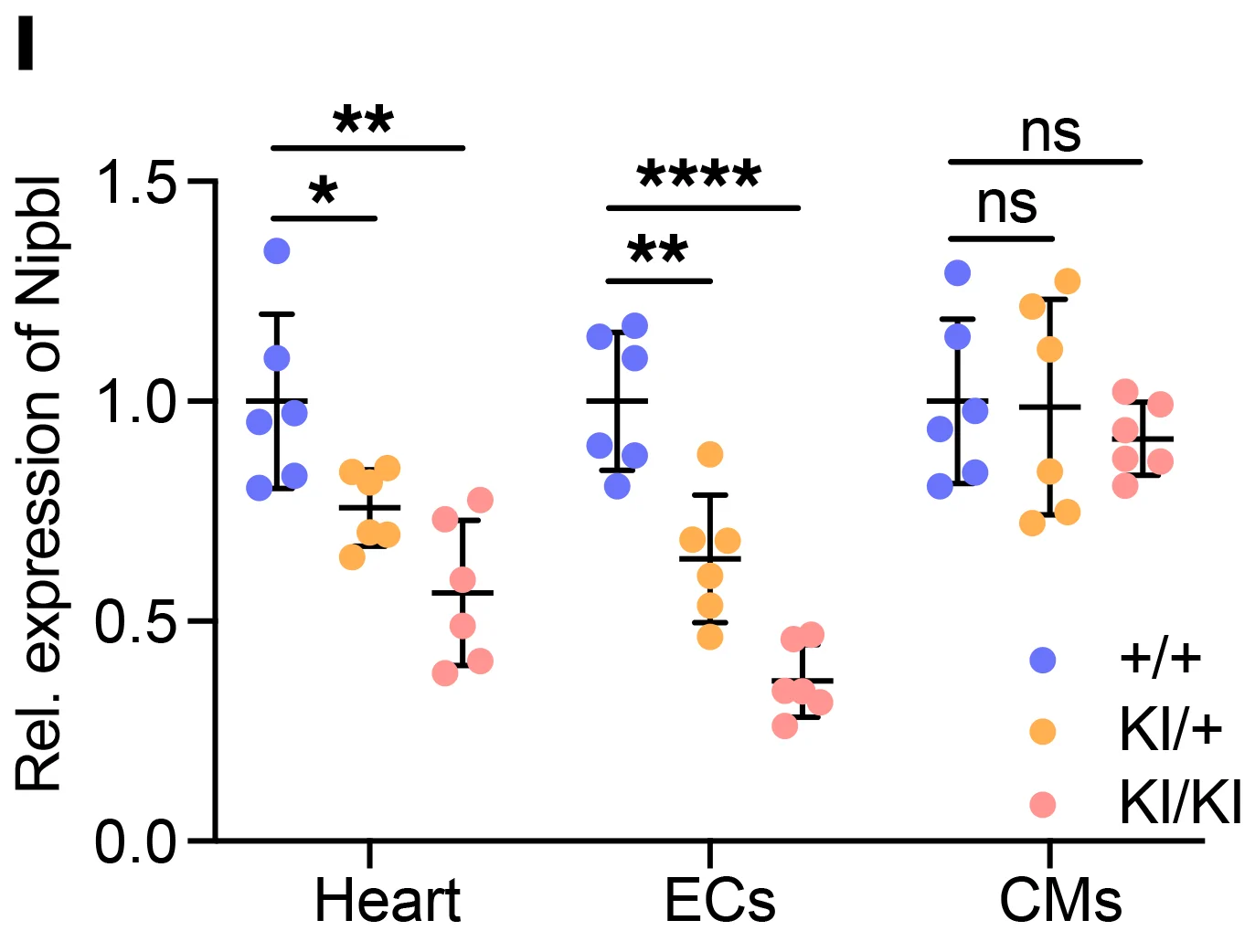# Corrigendum to Elevated microRNA-187 causes cardiac endothelial dysplasia to promote congenital heart disease through inhibition of NIPBL

**DOI:** 10.1172/JCI211195

**Published:** 2026-09-15

**Authors:** Chao Li, Zizheng Tan, Hongdou Li, Xiaoying Yao, Chuyue Peng, Yue Qi, Bo Wu, Tongjin Zhao, Chentao Li, Jianfeng Shen, Hongyan Wang

Original citation: *J Clin Invest*. 2025;135(1):e178355. https://doi.org/10.1172/JCI178355

Citation for this corrigendum: *J Clin Invest*. 2026;136(18):e211195. https://doi.org/10.1172/JCI211195

The authors recently identified errors in the raw data in Figure 4I for the NIPBL mRNA expression values for WT cardiomyocytes that occurred due to a copy-and-paste error. The corrected figure is shown below. In addition, a copy-and-paste error was identified in the raw data for Supplemental Figure 5C, which has been corrected with the original heart organoid volume data. The HTML and PDF versions of the paper, the supplemental data file, and the Supporting Data Values file have been updated.

The authors regret the errors.

## Figures and Tables

**Figure 4I:**