# Supplementary material for: Elevated microRNA-187 causes cardiac endothelial dysplasia to promote congenital heart disease through inhibition of NIPBL
Source: J Clin Invest. 2024 Nov 25;135(1):e178355. doi: 10.1172/JCI178355 (PMC11684815; doi:10.1172/JCI178355)
Supplement: Supplemental data [file jci-135-178355-s115.pdf]

Elevated microRNA-187 causes cardiac endothelial dysplasia to promote congenital heart disease through inhibition of NIPBL

Chao Li [1,2,#], Zizheng Tan[2,#], Hongdou Li [1,2], Xiaoying Yao[1], Chuyue Peng [1,2], Yue Qi[1,2], Bo Wu [3], Tongjin Zhao [2], Chentao Li [4], Jianfeng Shen [5,*] and Hongyan Wang [1,2,3,*]

1. Obstetrics and Gynecology Hospital, State Key Laboratory of Genetic Engineering, Children's Hospital, Fudan University, Shanghai 200438, China.

2. Shanghai Key Laboratory of Metabolic Remodeling and Health, Institute of Metabolism and Integrative Biology, Fudan University, Shanghai 200438, China

3. Prenatal Diagnosis Center of Shenzhen Maternity & Child Healthcare Hospital, Shenzhen 518028, China

4. Shanghai Medical College, Fudan University, Shanghai 200032, China

5. Department of Ophthalmology, Ninth People's Hospital, Shanghai JiaoTong University School of Medicine, Shanghai, 200025 China.

[#] These authors contributed equally.

*Correspondence should be addressed to Hongyan Wang (wanghy@fudan.edu.cn) and Jianfeng Shen (jfshen@shsmu.edu.cn), Tel. & Fax: 86-21-31246611

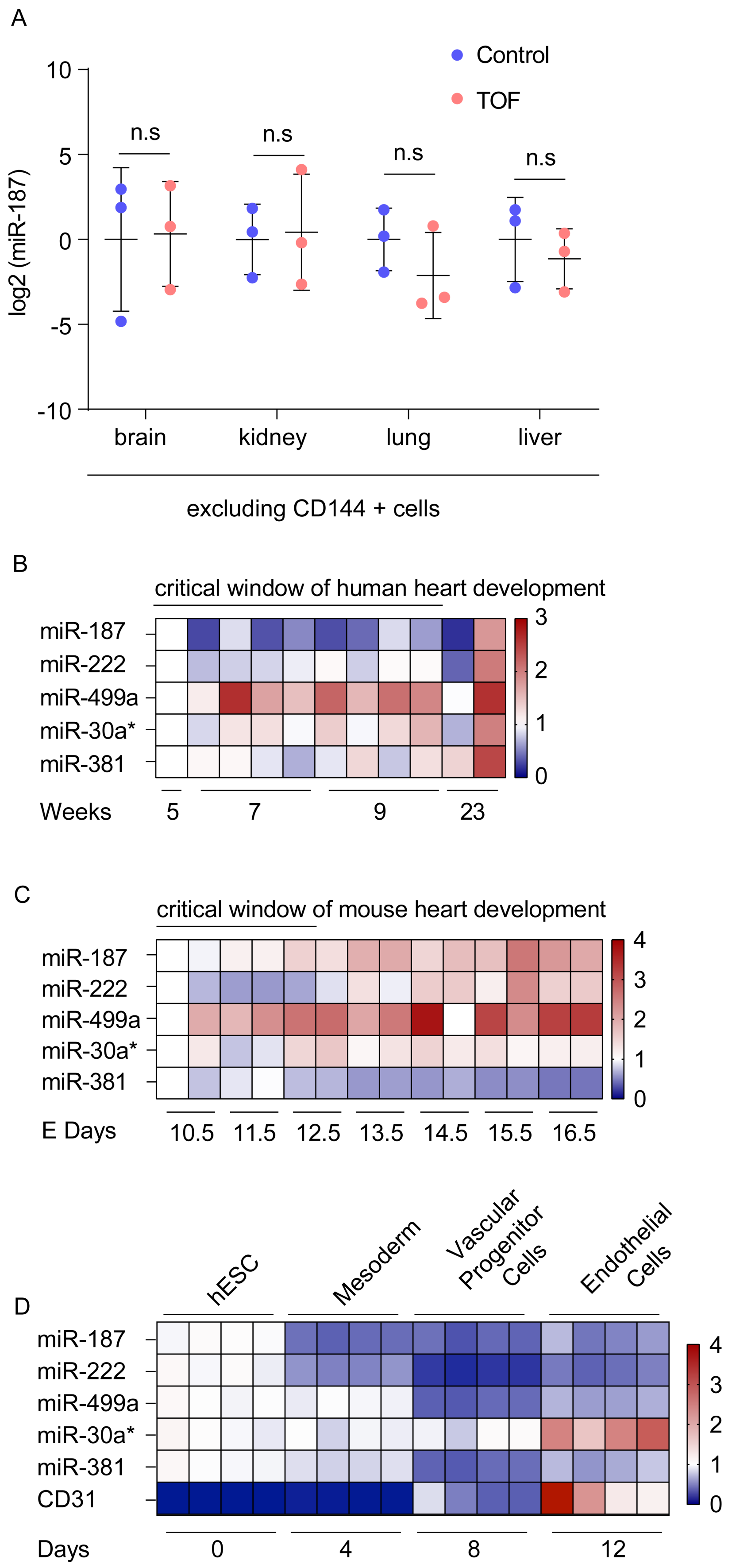

Figure S1. The expression of miR-187 in other tissues of fetuses with TOF. (A) RT-qPCR

analysis of miR-187 levels in the brains, kidneys, livers, and lungs excluding CD144 positive cells (n=3) of aborted fetuses with TOF and normal controls. U6 was used as an internal control. Data are shown as means ± SD. ns $P > 0.05$. **(B-D)** Heatmap representing the levels of differentially expressed miRNAs screened by microarray analysis in TOF patients **(B)**, mouse heart development using datasets GSE105834, GSE82960, GSE105910, GSE8604, GSE2822, GSE892942, and GSE101175 **(C)**, and differentiation of hESCs into ECs by RT-qPCR (n=4) **(D)**. Significance was determined by 1-way ANOVA (A).

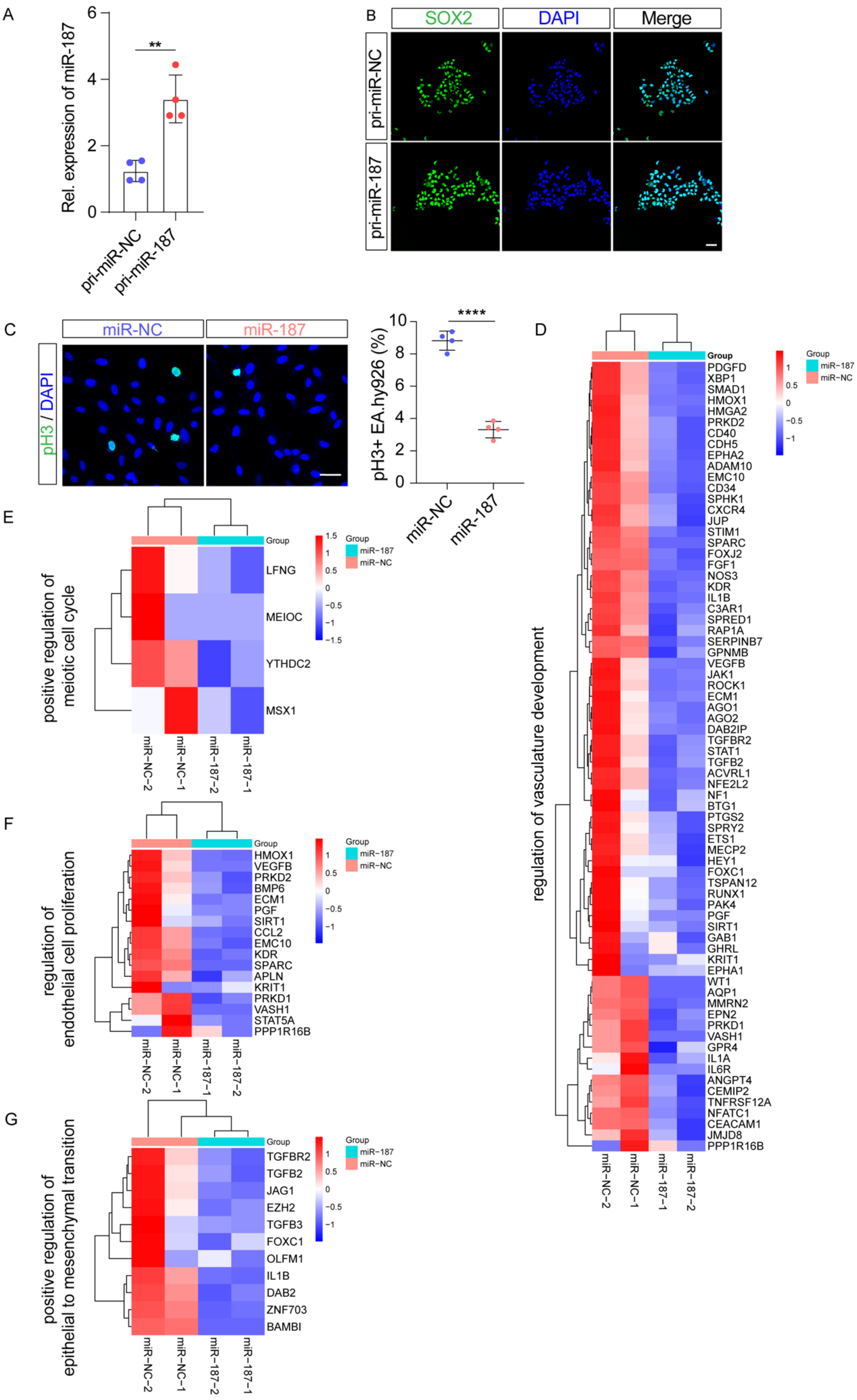
A
Rel. expression of miR-187
**
pri-miR-NC
pri-miR-187
B
SOX2
DAPI
Merge
pri-miR-NC
pri-miR-187
C
miR-NC
miR-187
pH3 / DAPI
pH3+ EA.hy926 (%)
****
miR-NC
miR-187
D
regulation of vasculature development
Group
miR−187
miR−NC
PDGFD
XBP1
SMAD1
HMOX1
HMGA2
PRKD2
CD40
CDH5
EPHA2
ADAM10
EMC10
CD34
SPHK1
CXCR4
JUP
STIM1
SPARC
FOXJ2
FGF1
NOS3
KDR
IL1B
C3AR1
SPRED1
RAP1A
SERPINB7
GPNMB
VEGFB
JAK1
ROCK1
ECM1
AGO1
AGO2
DAB2IP
TGFBR2
STAT1
TGFB2
ACVRL1
NFE2L2
NF1
BTG1
PTGS2
SPRY2
ETS1
HEY1
MECP2
FOXC1
TSPAN12
RUNX1
PAK4
PGF
SIRT1
GAB1
GHRL
KRIT1
EPHA1
WT1
AQP1
MMRN2
EPN2
PRKD1
VASH1
GPR4
IL1A
IL6R
ANGPT4
CEMIP2
TNFRSF12A
NFATC1
CEACAM1
JMJD8
PPP1R16B
miR−NC−2
miR−NC−1
miR−187−1
miR−187−2
E
positive regulation of meiotic cell cycle
Group
miR−187
miR−NC
LFNG
MEIOC
YTHDC2
MSX1
miR−NC−2
miR−NC−1
miR−187−2
miR−187−1
F
regulation of endothelial cell proliferation
Group
miR−187
miR−NC
HMOX1
VEGFB
PRKD2
BMP6
ECM1
PGF
SIRT1
CCL2
EMC10
KDR
SPARC
APLN
KRIT1
PRKD1
VASH1
STAT5A
PPP1R16B
miR−NC−2
miR−NC−1
miR−187−1
miR−187−2
G
positive regulation of epithelial to mesenchymal transition
Group
miR−187
miR−NC
TGFBR2
TGFB2
JAG1
EZH2
TGFB3
FOXC1
OLFM1
IL1B
DAB2
ZNF703
BAMBI
miR−NC−2
miR−NC−1
miR−187−2
miR−187−1

Figure S2. Construction of stable H9 cell lines expressing miR-187, NIPBL or scramble. **(A, B)** RT-qPCR analysis of miR-187 level (n=4) **(A)** and SOX2-immunofluorescence staining for pluripotency marker SOX2 (green) **(B)** in hESCs infected with lenti-pri-miR-187 or control virus. **(C)** Immunostainings for pH3 (green, left) and quantification show the number of mitotic mocks in EA.hy926 cells (n=4) (right). **(D-G)** Heat map showing expression changes of representative genes of regulation of vasculature development **(D)**, positive regulation of meiotic cell cycle **(E)**, regulation of endothelial cell proliferation **(F)** and positive regulation of epithelial to mesenchymal transition **(G)** with scramble and downregulation in ectopic expression of miR-187 EA.hy926 cells. The scale bars in (B) and (C) are 50 μm and 100 μm, respectively. DAPI was used for nuclear staining (blue). Data are shown as means ± SD. $^{**}P < 0.01$, $^{****}P < 0.0001$. Significance was determined by 2-tailed t test (A and C).

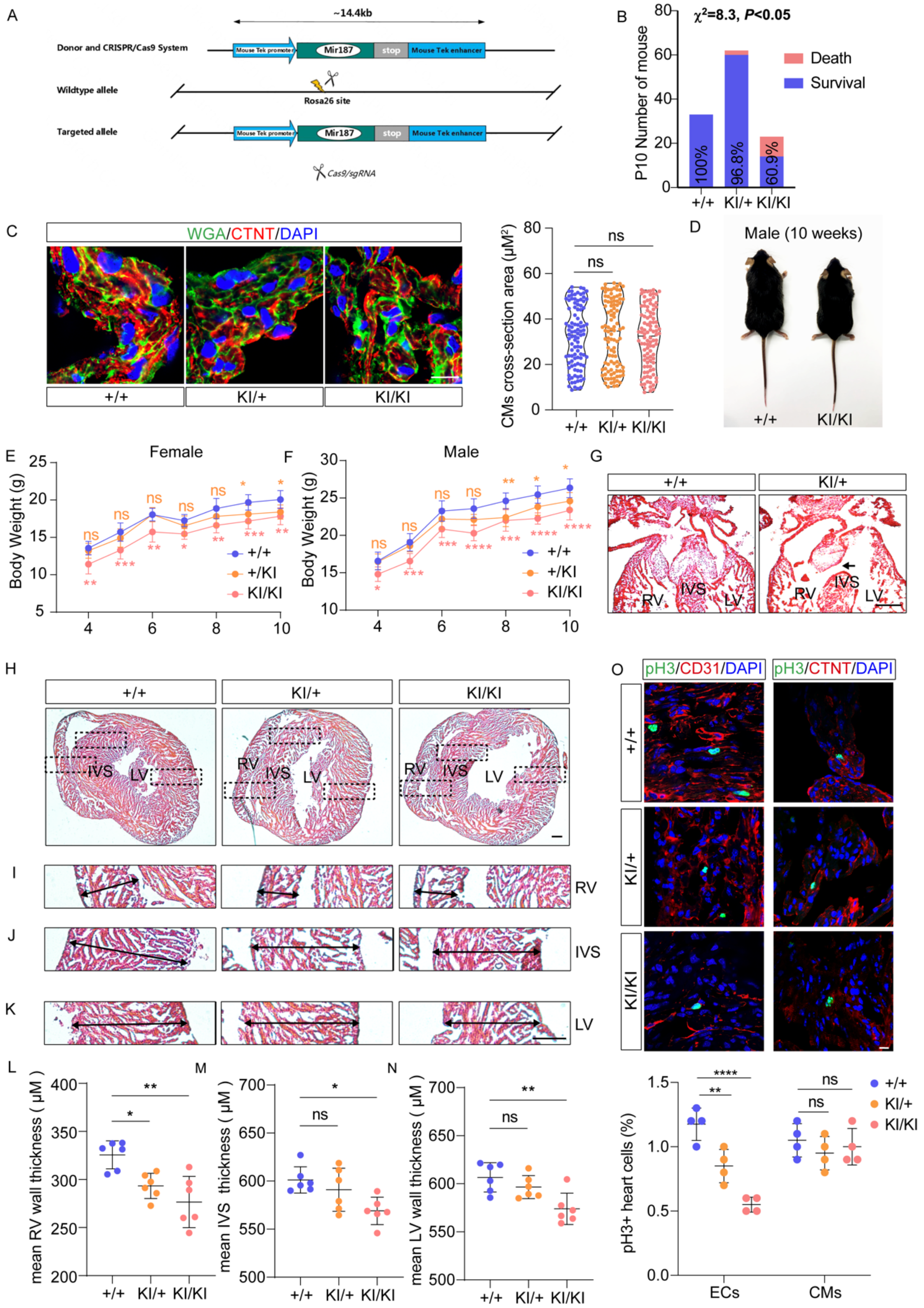

Figure S3. Endothelial-specific mmu-miR-187 knock-in mice. (A) Schematic illustration of strategy for knocking in TeK-mir-187 at the rosa26 locus. **(B)** The number of surviving and deceased normal controls and miR-187 KI mice after 10 days of birth. The survival rate is indicated in a specific column. Chi-square analysis is used to test whether the birth rate of miR-187 mice meets a 1:2:1 ratio. **(C)** Immunofluorescence staining of WGA and CTNT,

cardiac muscle cell marker (left) in heart sections from P0 neonatal mice and quantification (right) of the cross-section area of CMs (n=100). **(D)** Homozygous miR-187-KI mice are smaller in size than WT (+/+) littermates at week 10. **(E, F)** Homozygous female **(E)** and male **(F)** weighed less than WT from week 4 to week 10 (n=6). **(G)** H&E-stained heart sections from heterozygous mir-187 KI and control mice, displaying human CHD-like phenotypes, such as the control heart shows a normal septum; A mir-187 KI littermate of the animal in shows VSD at P0. **(H-K)** Double head arrows indicate the thickness of the compact myocardium of RV **(I)**, IVS **(J)** and LV **(K)**. **(L-N)** Quantification of the thickness of compact myocardium of RV **(L)**, IVS **(M)** and LV **(N)**. **(O)** Immunostainings for pH3, CD31 (endothelial cells marker) and CTNT (cardiomyocyte marker) show the number of heart mitotic cell at P0. DAPI was used for nuclear staining. The scale bars in (C), (G-K) and (O) are 20 μm, 200 μm and 10 μm, respectively. Data are shown as means ± SD. ns $P > 0.05$, $*P < 0.05$, $**P < 0.01$, $***P < 0.001$. Significance was determined by 1-way ANOVA (C, L-O), 2-way ANOVA (E and F) and Pearson's χ2-test (B).

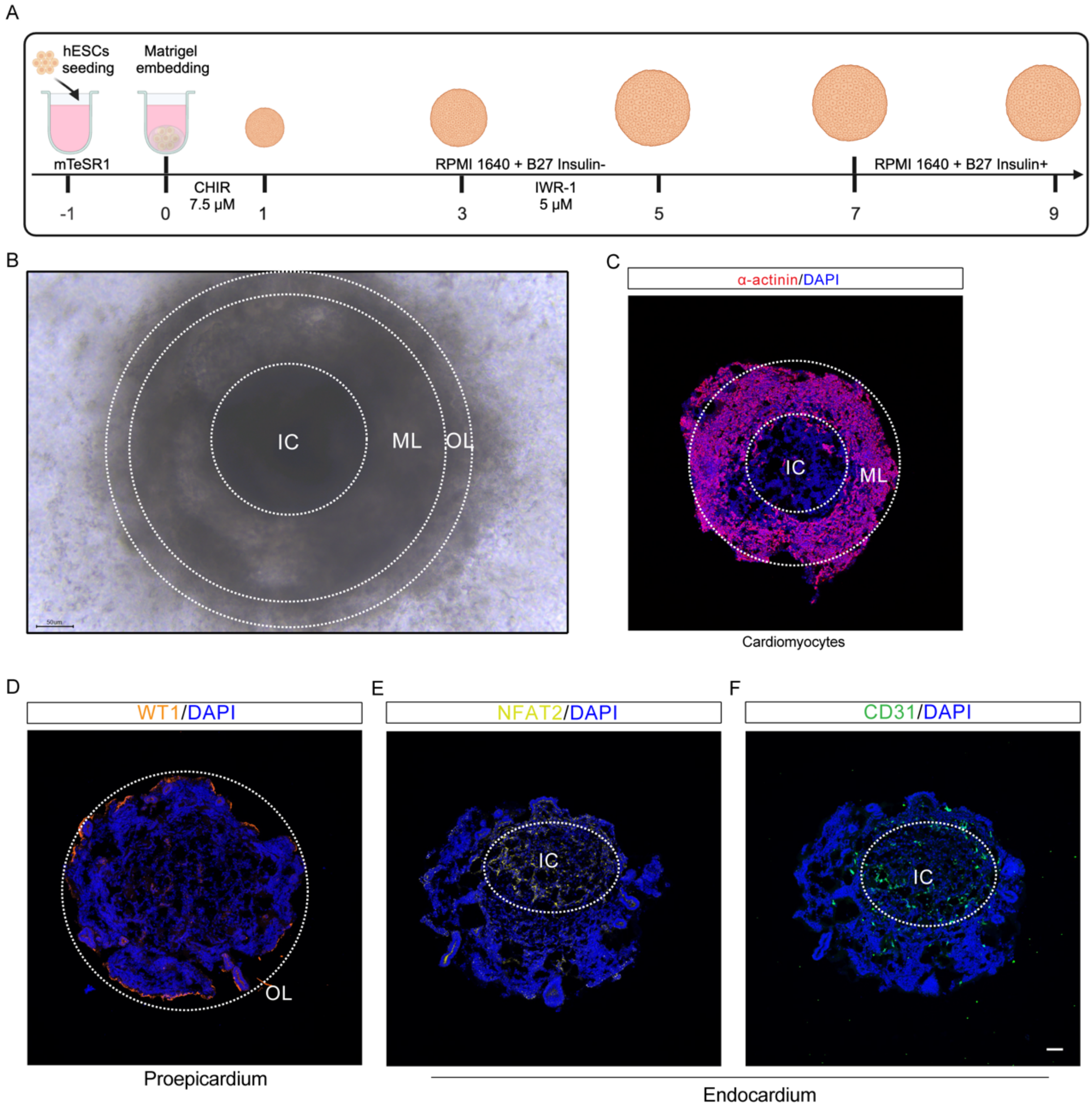

Figure S4. Human heart-forming organoids are composed of a myocardial layer (ML) lined by endocardial inner core (IC) and surrounded by proepicardial outer layer (OL)

anlagen. **(A)** The protocol for HFO formation involved embedding hESC aggregates individually in Matrigel and differentiating them using CHIR and IWR-1. **(B)** A typical hESC-derived HFO forming three layers: IC, ML and OL. **(C-F)** A section stained for cardiomyocyte (α-actinin, **C**), proepicardium (WT1, **D**) and endocardium markers (NFAT2 and CD31, **E, F**) antibody. The scale bars in (B) and (C-F) are 50 µm and 100 µm, respectively.

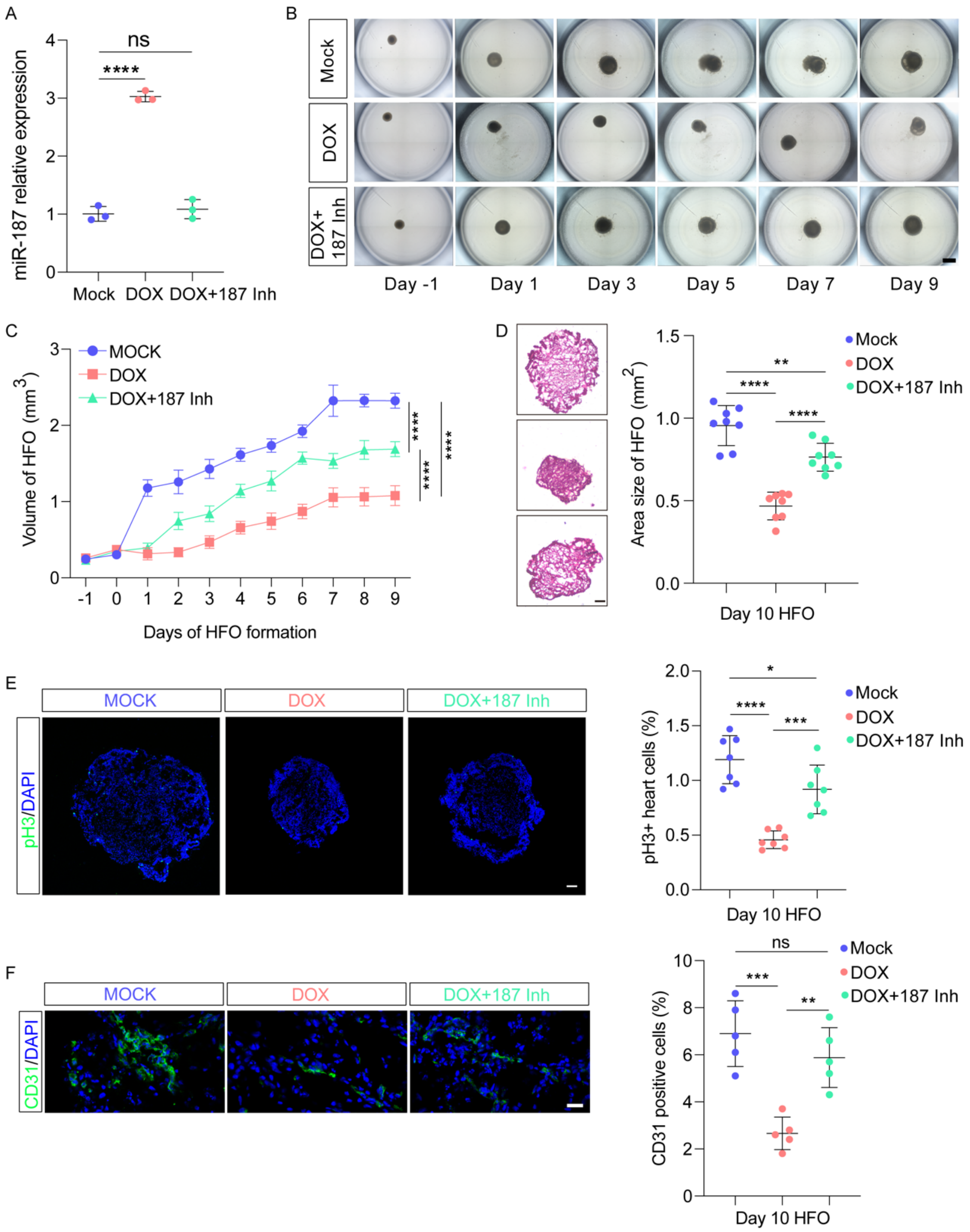

Figure S5. Doxorubicin distributed human heart-forming organoids endothelial cell differentiation and mitosis by increasing miR-187. (A) RT-qPCR detected miR-187 relative expression after DOX and DOX/miR-187 inhibitor treated in HFOs. **(B)** The figure shows the development of HFOs from day −1 until day 9 of mock, DOX and DOX/miR-187 inhibitor

differentiation. **(C)** Quantification of volume of HFOs (n=10) from day −1 until day 9 of mock, DOX and DOX/miR-187 inhibitor differentiation. **(D)** H&E staining (left) and quantification (n=8) (right) of area for mock, DOX and DOX/miR-187 inhibitor HFOs at day 10. **(E)** Immunostainings for pH3 (green, left) and quantification (n=8) (right) show the number of mitotic mock, DOX and DOX/miR-187 inhibitor HFO cells at day 10. **(F)** Representative immunofluorescence (green, left) and quantification (n=8) (right) staining of the endothelial cell marker CD31 in mock, miR-187 and miR-187/NIPBL HFO cells at day 10. DAPI was used for nuclear staining (blue). The scale bars in (B), (D, E) and (F) are 1 mm, 100 µm and 20 µm, respectively. Data are shown as means ± SD. ns $P > 0.05$, *$P < 0.05$, **$P < 0.01$, ****$P < 0.0001$. Significance was determined by 1-way ANOVA (A, D-F) and 2-way ANOVA (C).

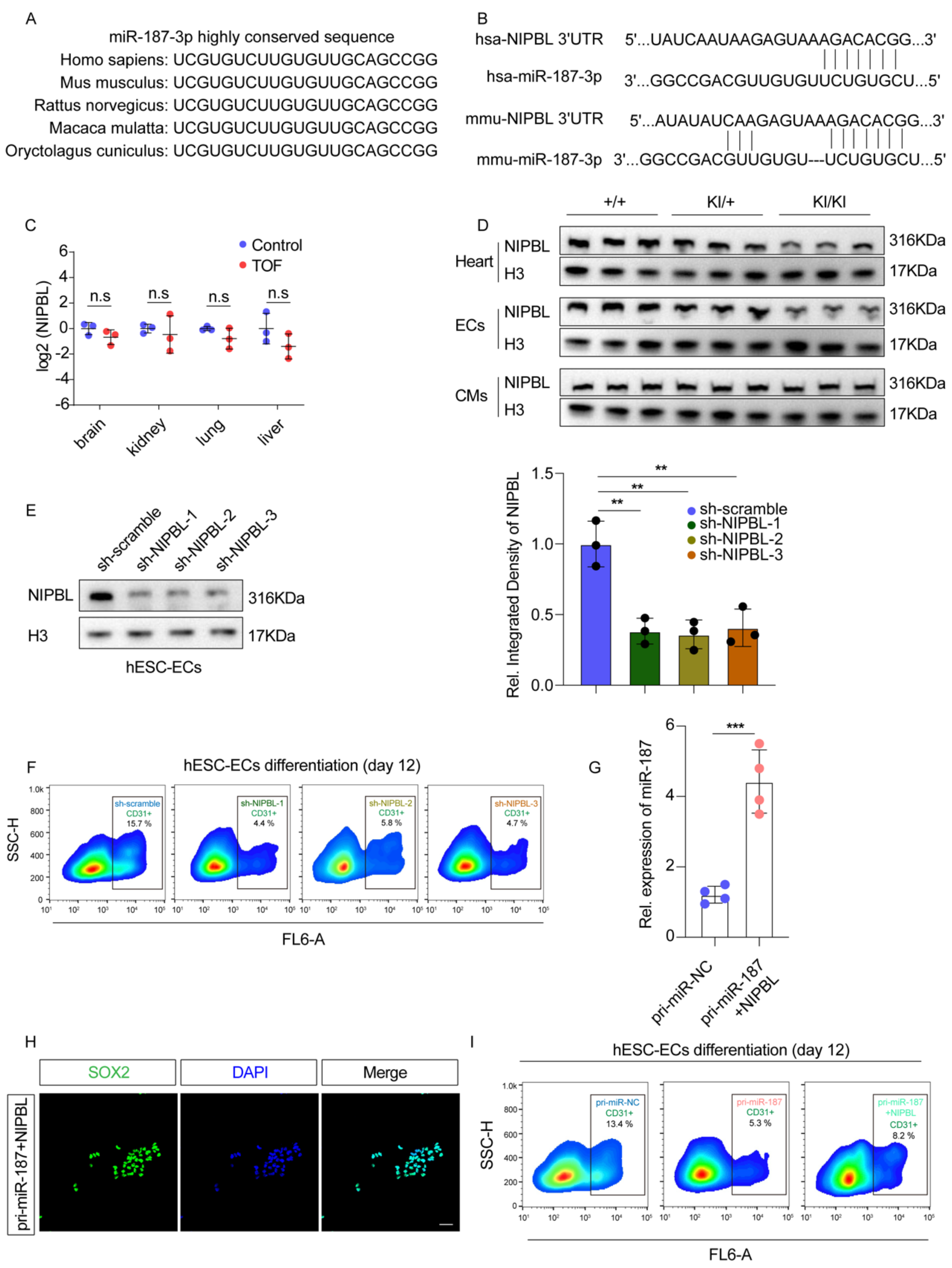

Figure S6. miR-187 disturbs endothelial differentiation. (A) The schematic depicts the conservation of the miR-187-3p sequence across various species. **(B)** The schematic illustrates the binding sequences of miR-187 and NIPBL 3'UTR in human and mouse. **(C)** RT-qPCR analyses of levels of NIPBL in brains, kidneys, livers and lungs of aborted fetuses with TOF (n=3) and normal controls (n=3). **(D)** Western blotting was performed to analyze the levels of Nipbl in whole hearts, cardiac endothelial cells, and cardiomyocytes of P0 mice with the indicated genotypes (n=3). H3 was used as a loading control. **(E)** Western blotting of NIPBL in hESC-ECs expressed sh-NIPBL-1, 2 and 3 or sh-scramble as indicated. H3 was used

as a loading control. **(F, I)** FACS analysis of CD31 positive cells in hESC-ECs infection with sh-NIPBL-1, 2, 3 (n=4) **(F)**, sh-scramble, pri-miR-187, pri-miR-187+NIPBL or scramble control by lentivirus (n=4) **(I)**. **(G, H)** RT-qPCR analysis of miR-187 level (n=4) **(G)** and SOX2-immunofluorescence staining for pluripotency marker SOX2 (green) **(H)** in hESCs infected with miR-187/NIPBL or control virus. Scale bars in **(H)** are 50 μm. Data are shown as means ± SD. ns $P > 0.05$, $**P < 0.01$, $***P < 0.001$. Significance was determined by 1-way ANOVA (C, E) and 2-tailed t test (G).

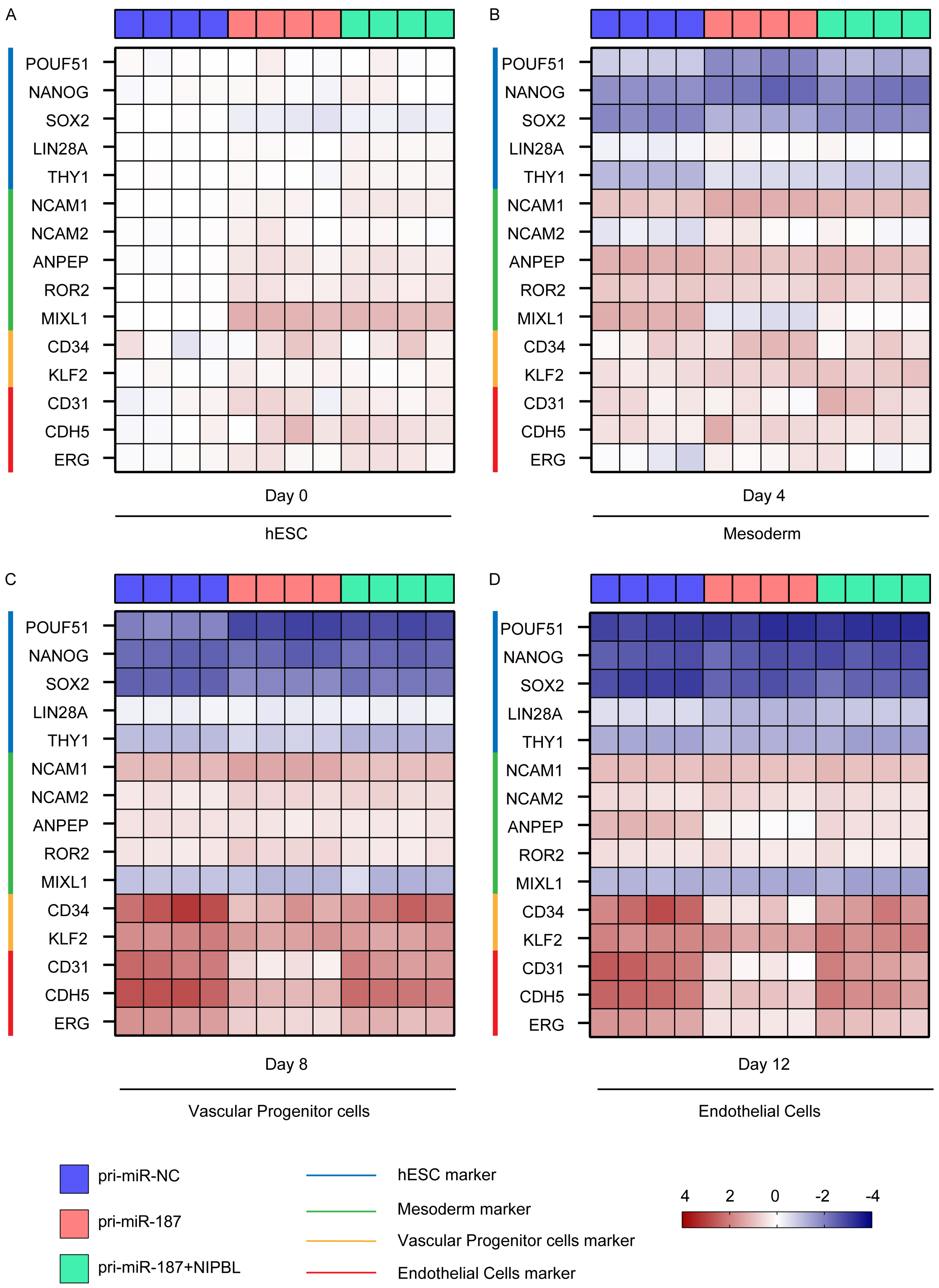

Figure S7. NIPBL recovered miR-187-mediated inhibition of endothelial differentiation. (A-D) Heat map of RT-qPCR analyses of expression levels of various markers for hESC **(A)**, mesoderm **(B)**, vascular progenitor cells **(C)** and mature endothelial cells **(D)** during differentiation from hESCs to endothelial cells (n=4).

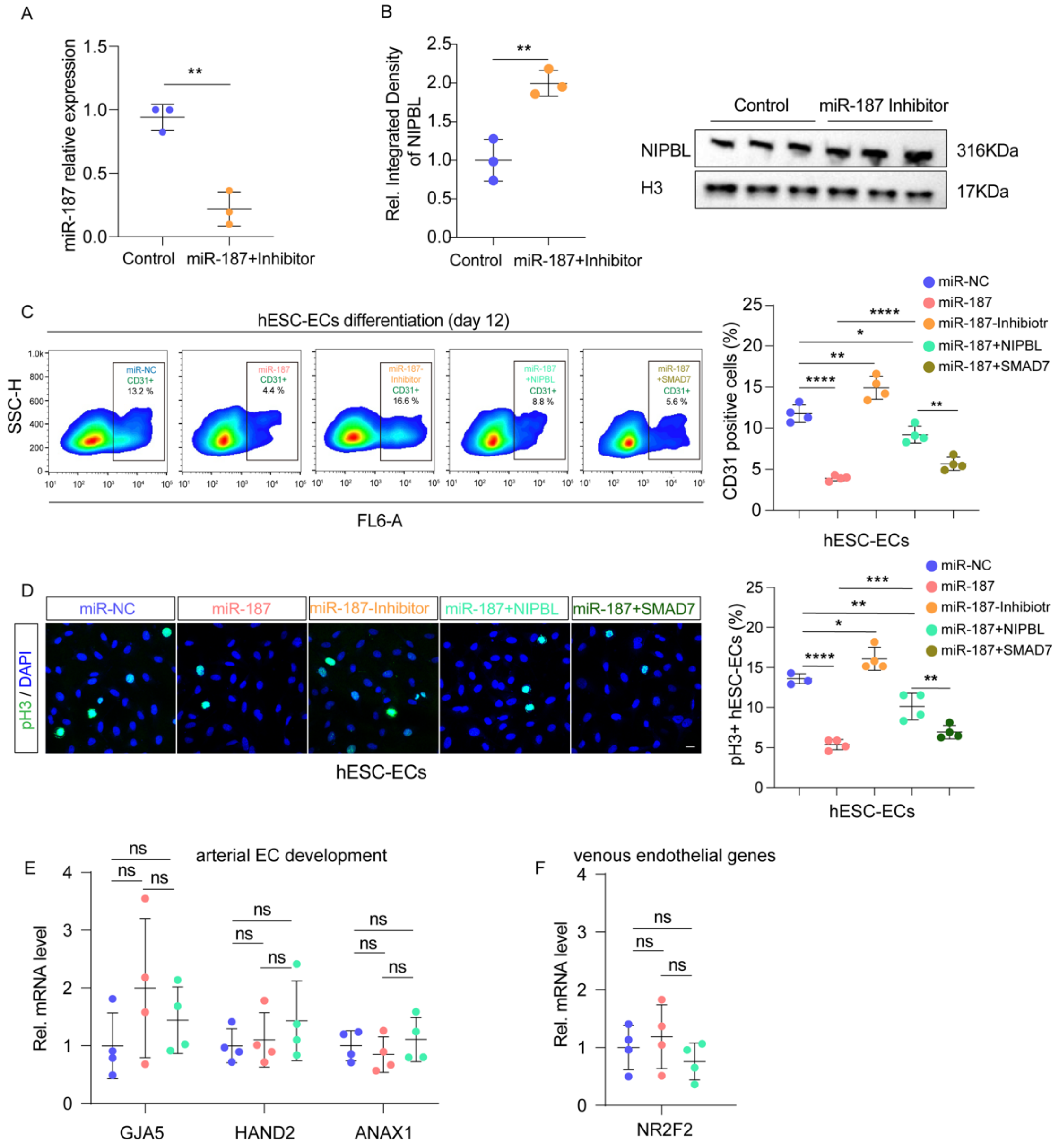

Figure S8. miR-187 inhibits endothelial cell differentiation and mitosis. (A,B) RT-qPCR and Western blotting were used to respectively measure the levels of miR-187 **(A)** and the protein expression of NIPBL **(B)** after adding a miR-187 inhibitor in hESC-ECs. **(C, D)** During hESC differentiation into hESC-ECs, miR-NC, miR-187, miR-187-inhibitor, miR-187/NIPBL, and miR-187/SMAD7 were overexpressed. FACS was used to quantify CD31-marked hESC-ECs as a measure of differentiation efficiency **(C)**. Immunofluorescence was performed to assess the mitotic capability of pH3-marked hESC-ECs **(D)**. **(E, F)** RT-qPCR analyses of expression levels of various markers for arterial EC development **(E)** and venous endothelial genes **(F)** (n=4). GAPDH was used as an internal control. The scale bars in (D) are 20 μm. Data are shown as means ± SD. ns $P > 0.05$, $*P < 0.05$, $**P < 0.01$, $***P < 0.001$,

****$P < 0.0001$. Significance was determined by 1-way ANOVA (C-F) and 2-tailed t test (A and B).

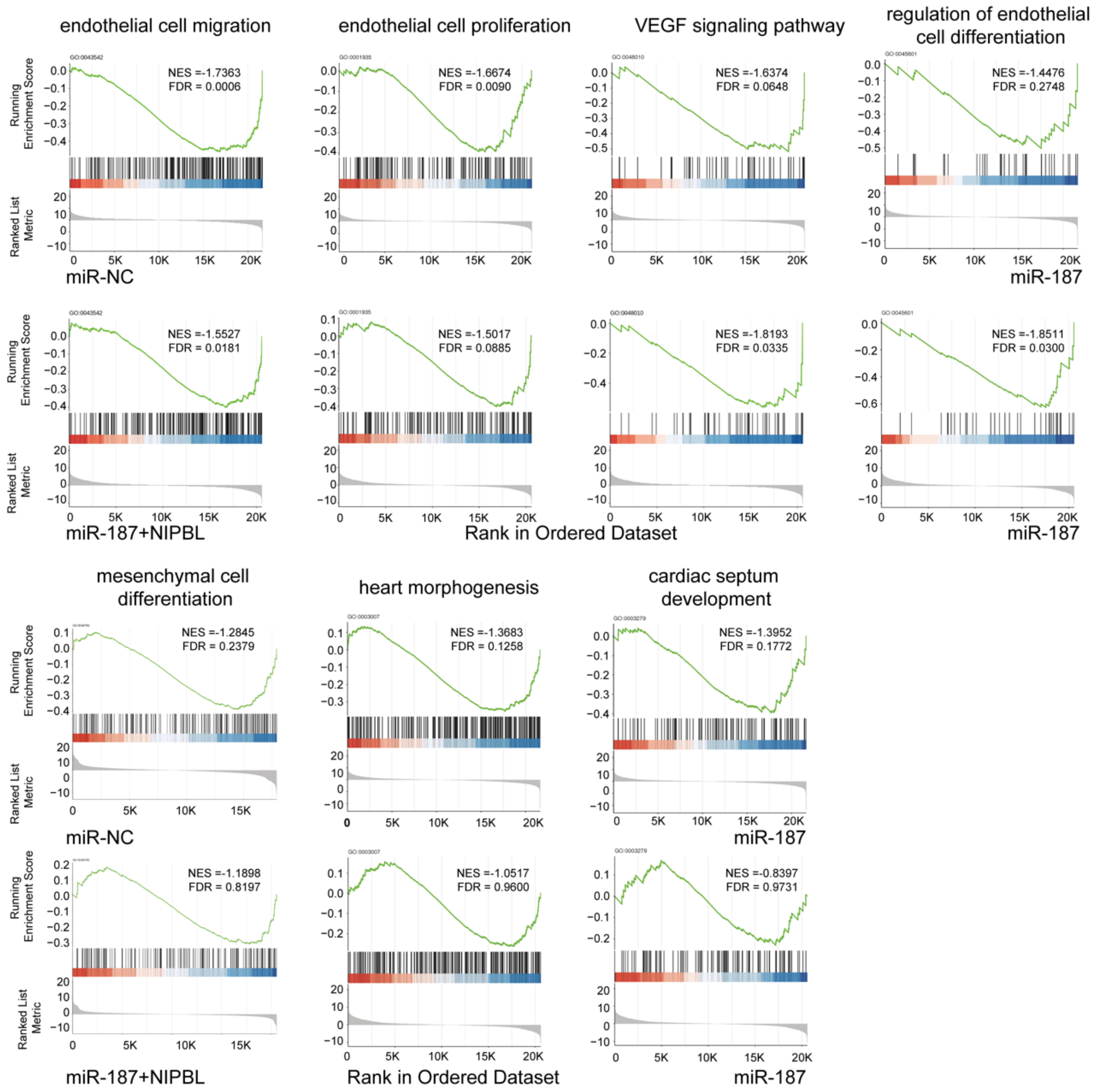

Figure S9. miR-187/NIPBL axis inhibits endocardial gene expression. Representative GSEA results for endothelial cell migration (GO:0043542), endothelial cell proliferation (GO:0001935), vascular endothelial growth factor receptor signaling pathway (GO:0048010), regulation of endothelial cell differentiation (GO:0045601), mesenchymal cell differentiation (GO:0048762), heart morphogenesis (GO:0003007) and cardiac septum development (GO:0003279) gene sets.

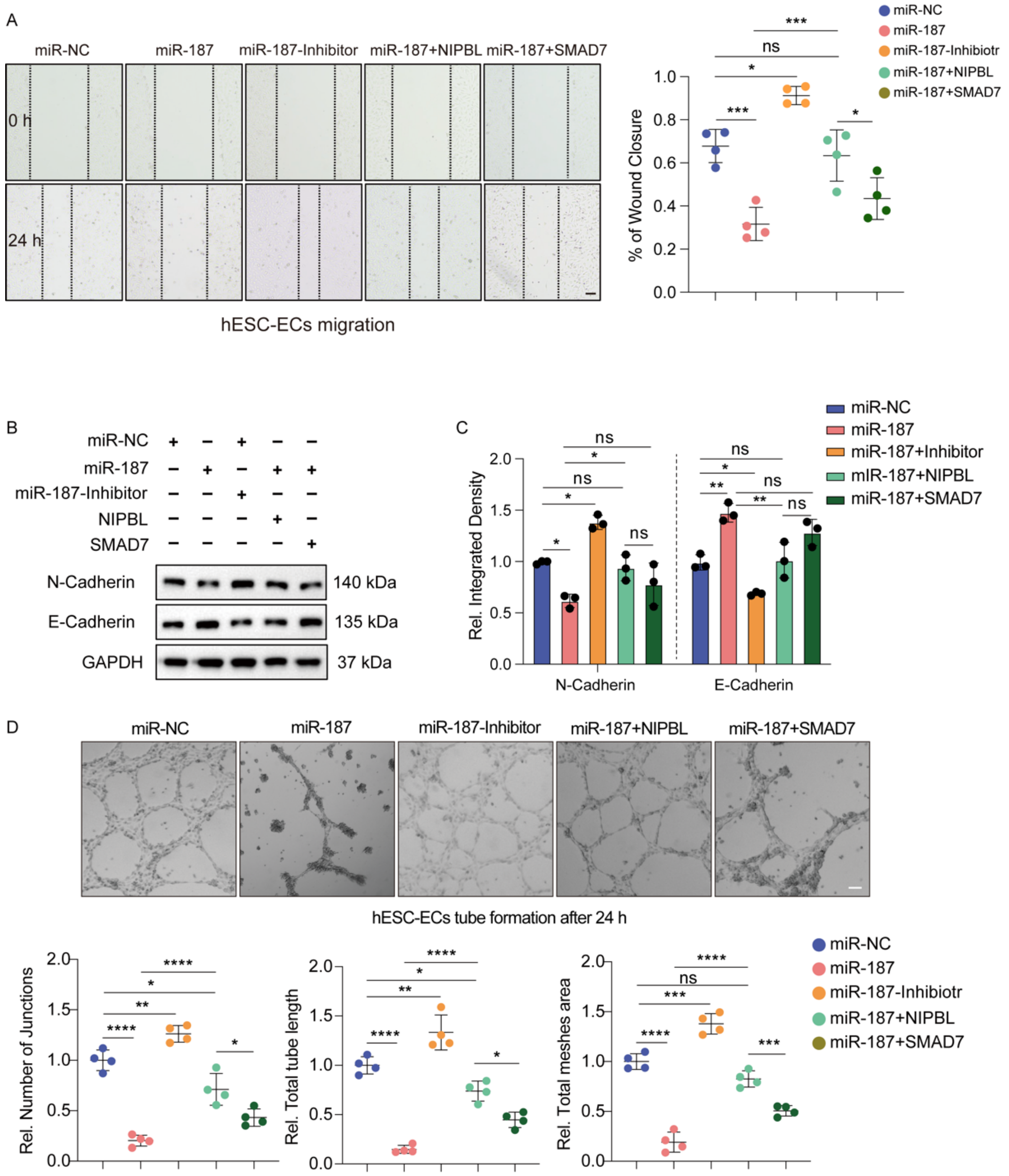

Figure S10. miR-187 inhibits endothelial cell migration, epithelial to mesenchymal transition and tube formation. (A-D) During hESC differentiation into hESC-ECs, miR-NC, miR-187, miR-187-inhibitor, miR-187/NIPBL, and miR-187/SMAD7 were overexpressed. **(A)** The migration ability was determined by the wound healing assays. The wound closure area was measured and quantified (n=4). **(B, C)**Western blotting analysis **(B)** and quantification **(C)** of protein levels of N-Cadherin and E-Cadherin (n=3). GAPDH was used as a loading control. **(D)** Tube formation assays revealed a marked reduction in tube formation by hESC-ECs transfected with miR-187 mimic (upper), quantified by assessing the number of tubes, nodes, and meshes (bottom) (n=4). The scale bars in **(A, D)** are and 100 μm. Data are shown as means ± SD. ns P > 0.05, **P* < 0.05, ***P* < 0.01, ****P* < 0.001, *****P* < 0.0001. Significance was determined by 1-way ANOVA (A, C and D).

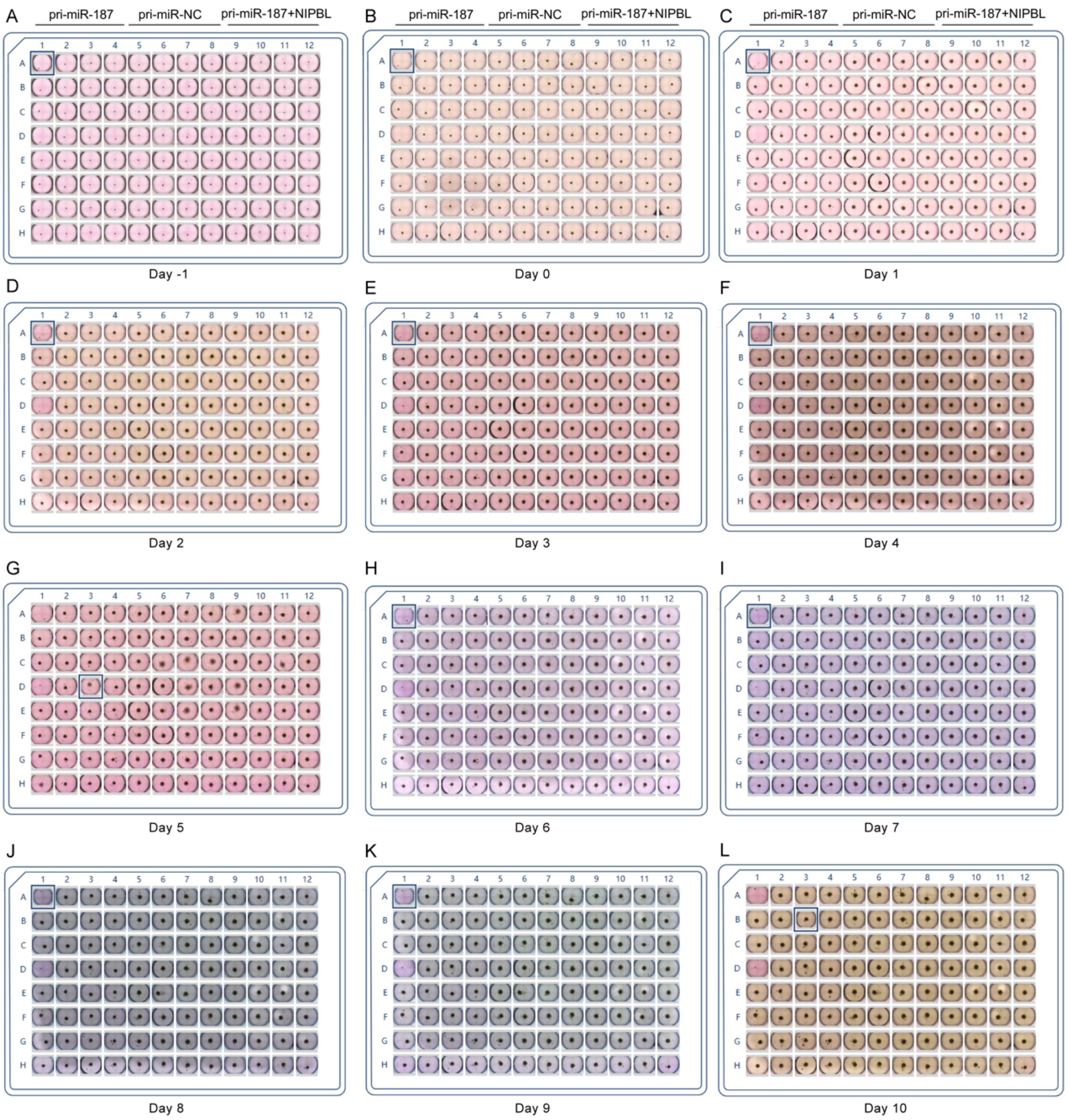

Figure S11. NIPBL recovers delaying HFO formation induced by miR-187. (A-L) The figure shows the development of HFOs from day −1 to day 10 of mock **(A-L)**, miR-187 and miR-187/NIPBL differentiation.

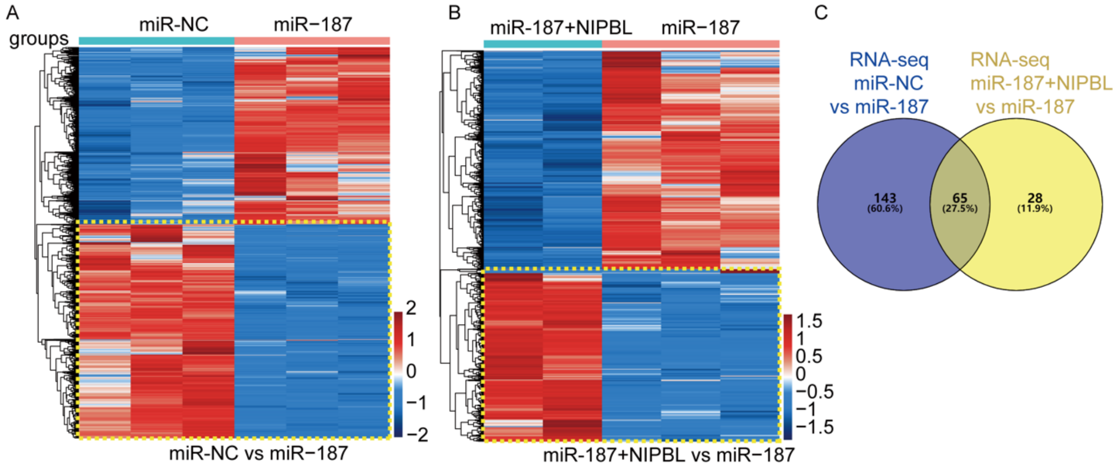

Figure S12. RNA-Seq Screening of Differentially Expressed Genes in hESC-ECs

Influenced by miR-187 and NIPBL. (A, B) Heat map of RNA-seq analyses of differentially expressed genes for pri-miR-NC-hESC-ECs vs pri-miR-187-hESC-ECs **(A)** and pri-miR-187/NIPBL-hESC-ECs vs pri-miR-187-hESC-ECs **(B)**. **(C)** Schematic illustration of the screening approach for downstream genes of miR-187/NIPBL axis using miR-NC-hESC-ECs, miR-187-hESC-ECs and miR-187/NIPBL-hESC-ECs RNA-seq.

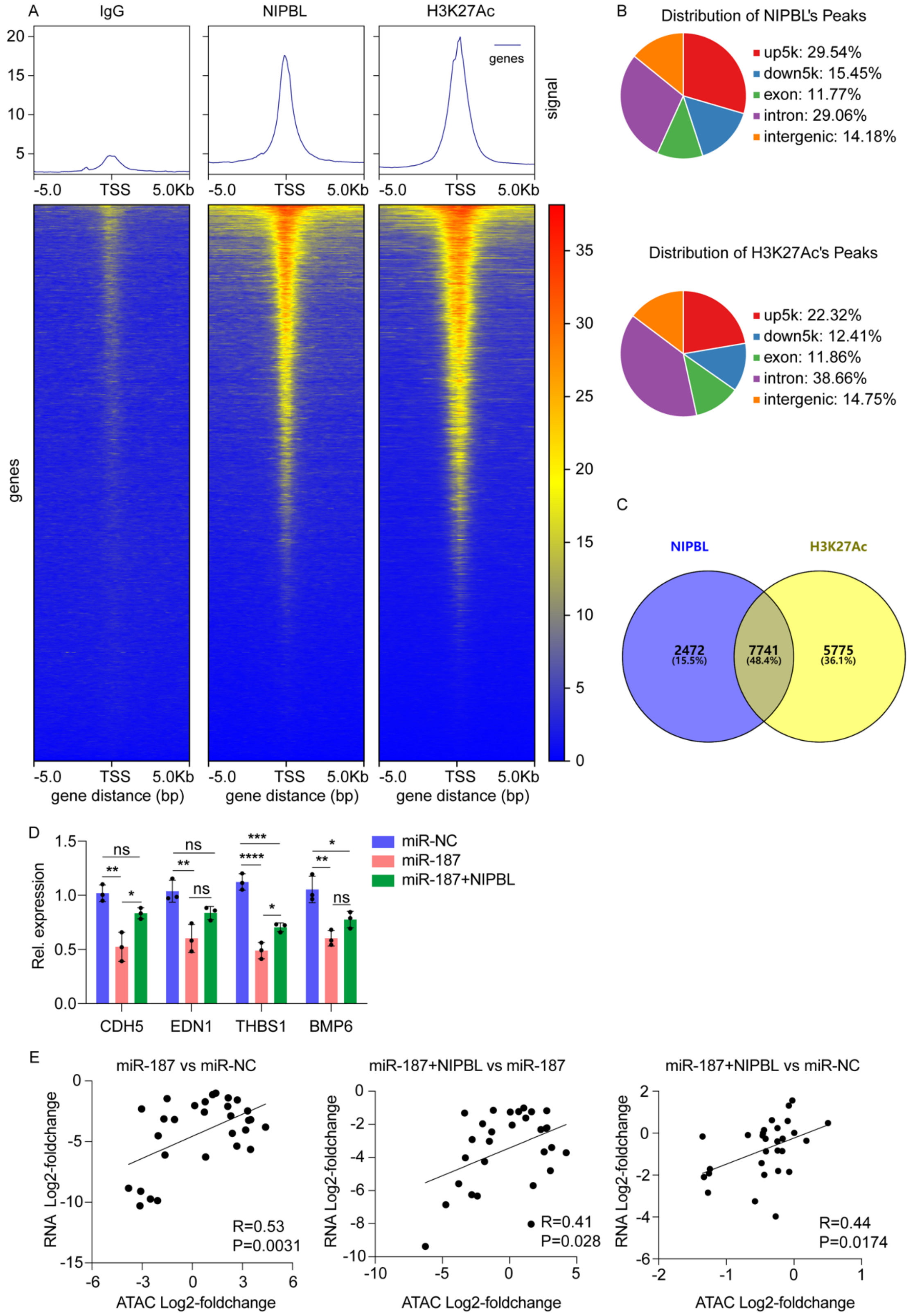

Figure S13. miR-187/NIPBL axis inhibits endocardial gene chromatin accessibility. (A, B) Density heatmaps **(A)** and distributions **(B)** of IgG-CUT&Tag, NIPBL-CUT&Tag and H3K27Ac-CUT&Tag in hESC-ECs. **(C)** Schematic illustration of the screening approach for downstream genes of miR-187/NIPBL axis using NIPBL, H3K27Ac CUT&TAG-seq. **(D)** Gene expression levels were detected by RNA-qPCR after exogenous miR-NC, miR-187, miR-187+NIPBL transfection in hESC-ECs. **(E)** The scatter plot depicts the correlation between RNA-seq and ATAC-seq data for 29 screened genes in hESC-ECs. Correlation coefficients and *p*-values are annotated in the figure. Data are shown as means ± SD. **P* < 0.05, ***P* < 0.01, ****P* < 0.001. Significance was determined by 1-way ANOVA (D).

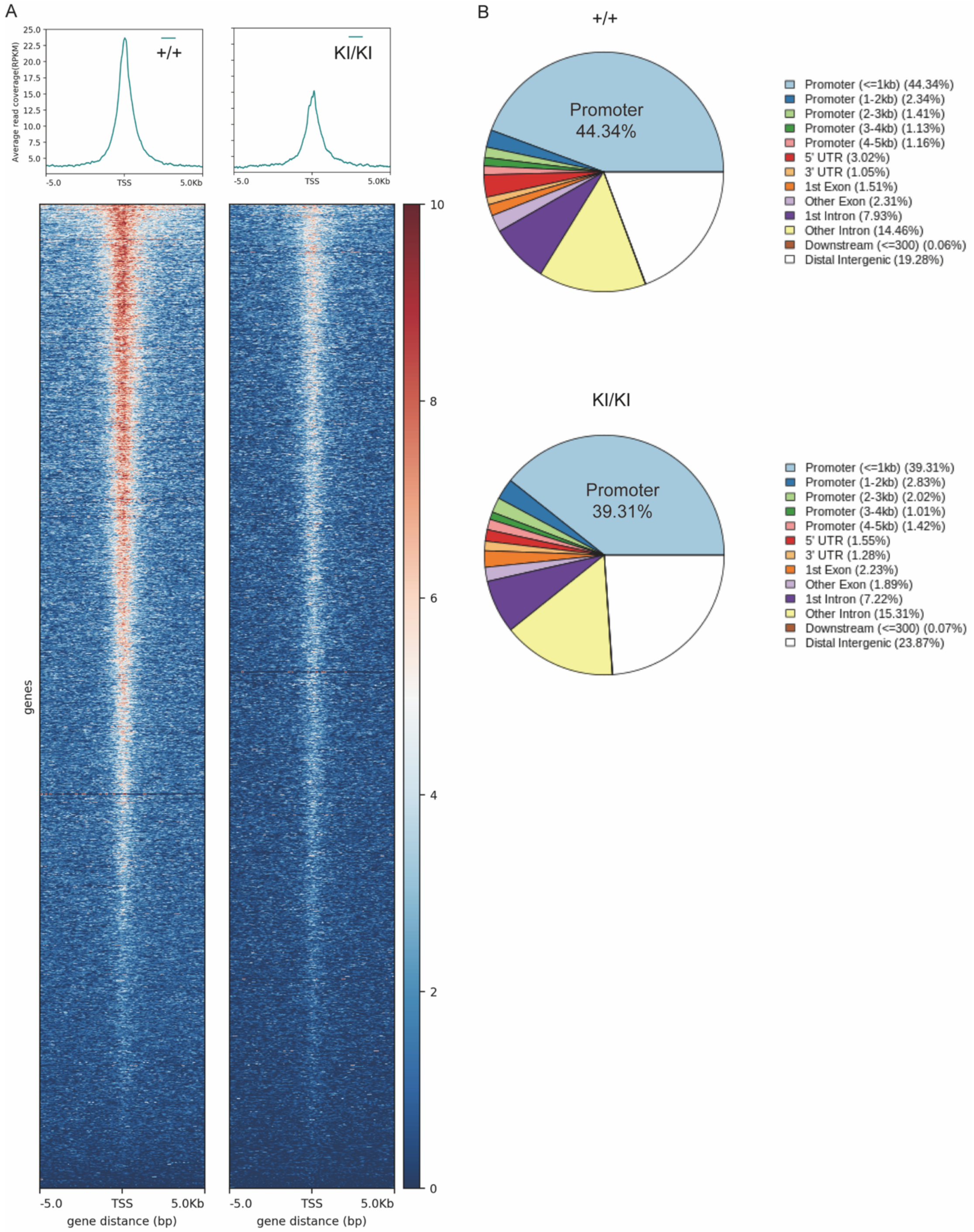

Figure S14. Global chromatin accessibility changes in the endocardium of miR-187-KI mice. (A) Average normalized ATAC-Seq signal intensity for all peaks changing in accessibility in WT and miR-187-KI mice (upper). Heatmap of signal distribution around ATAC-Seq peak summits for the same peaks (bottom). **(B)** Pie charts showing the distribution of genomic features among all peaks in the endocardium cardiac endothelial cells of miR-187-KI mice.

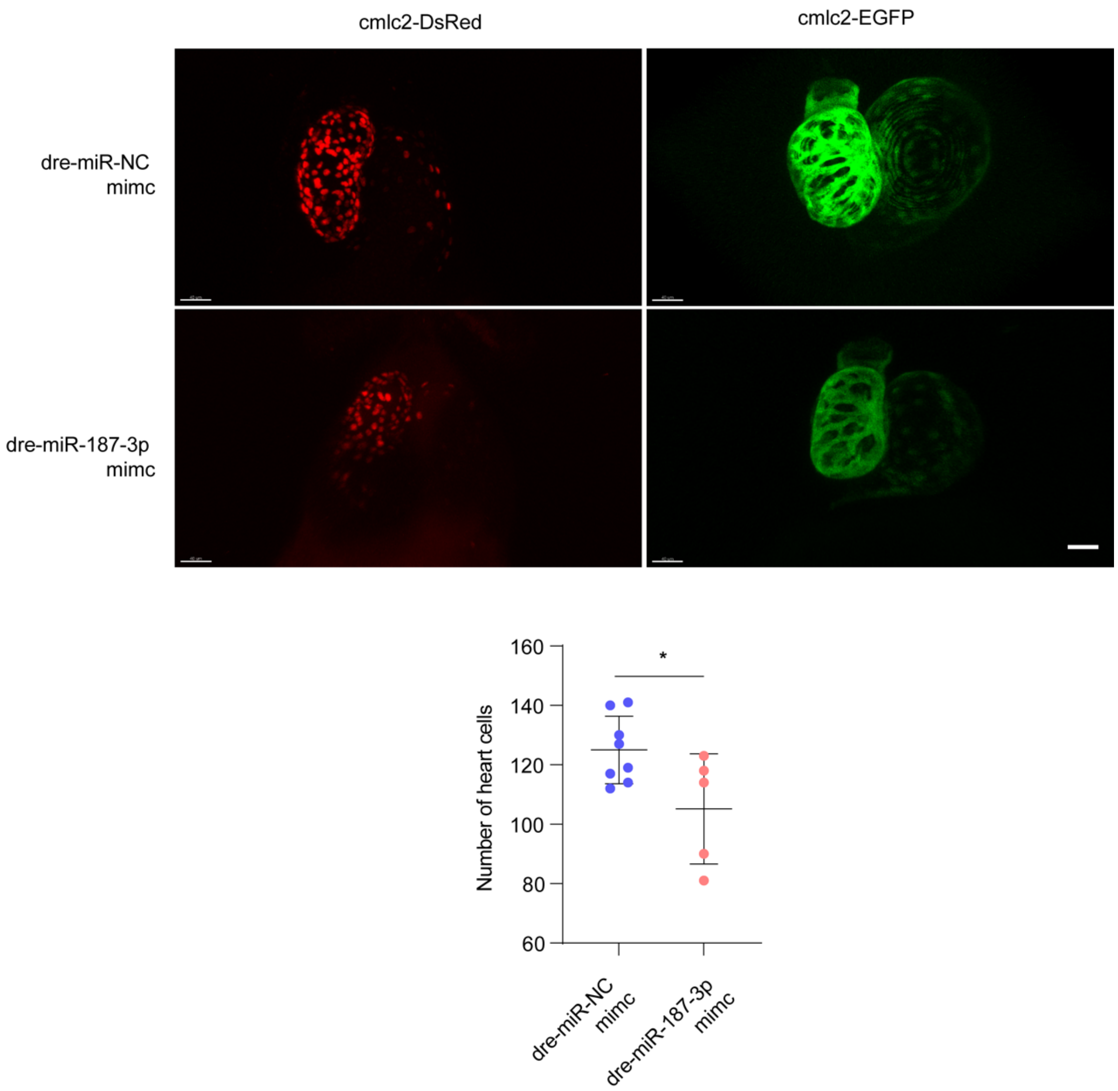

Figure S15. dre-miR-187 reduces the number of heart cells in zebrafish. Images (upper) and quantification (bottom) of hearts cells in cmlc2-DsRed and cmlc2-EGFP zebrafish at 72 hpf. Significance was determined by 2-tailed t test.

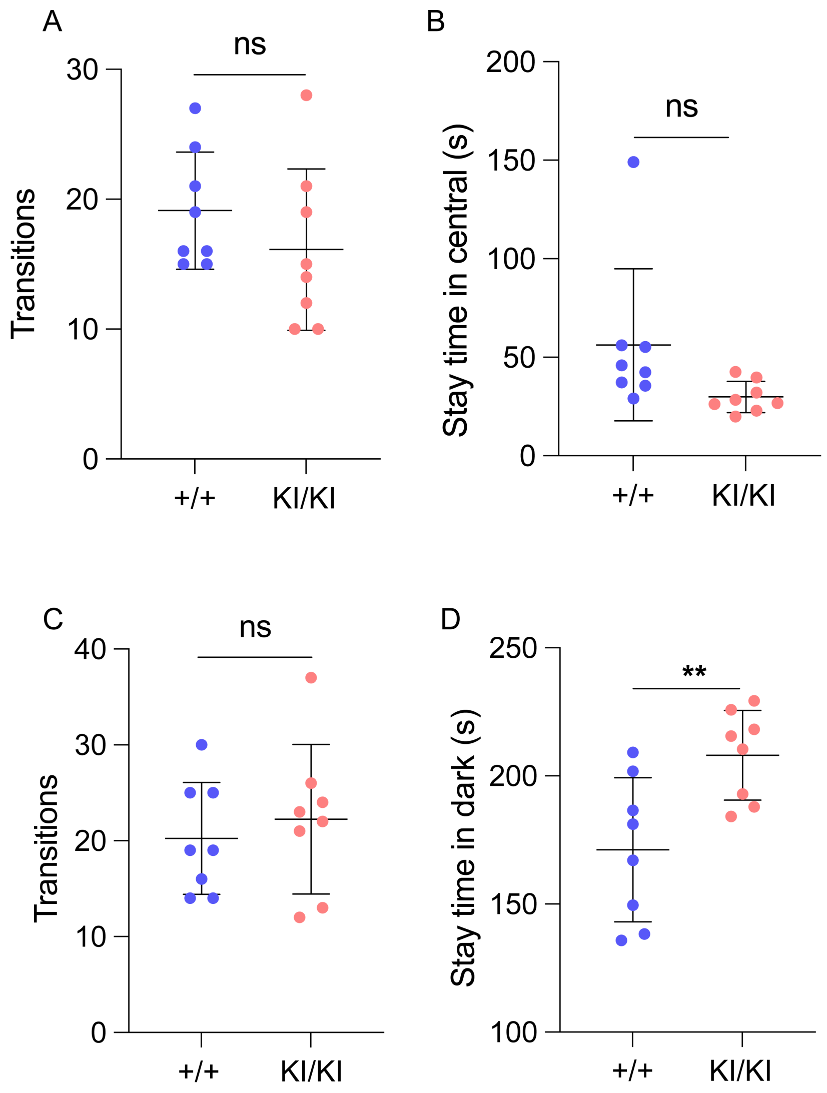

Figure S16. miR-187 KI mice display anxiety-like behavior. (A, B) Transitions of mice between peripheral and central zones **(A)** and duration of time spent in the central zone during the open field test **(B)**. **(C, D)** Transition frequency between light and dark compartments **(C)** and time spent in the dark chamber **(D)**. Significance was determined by 2-tailed t test (A-D).
