# Supplementary material for: Elevated microRNA-187 causes cardiac endothelial dysplasia to promote congenital heart disease through inhibition of NIPBL
Source: J Clin Invest. 2024 Nov 25;135(1):e178355. doi: 10.1172/JCI178355 (PMC11684815; doi:10.1172/JCI178355)

Full unedited blots for Figure 4G

NIPBL

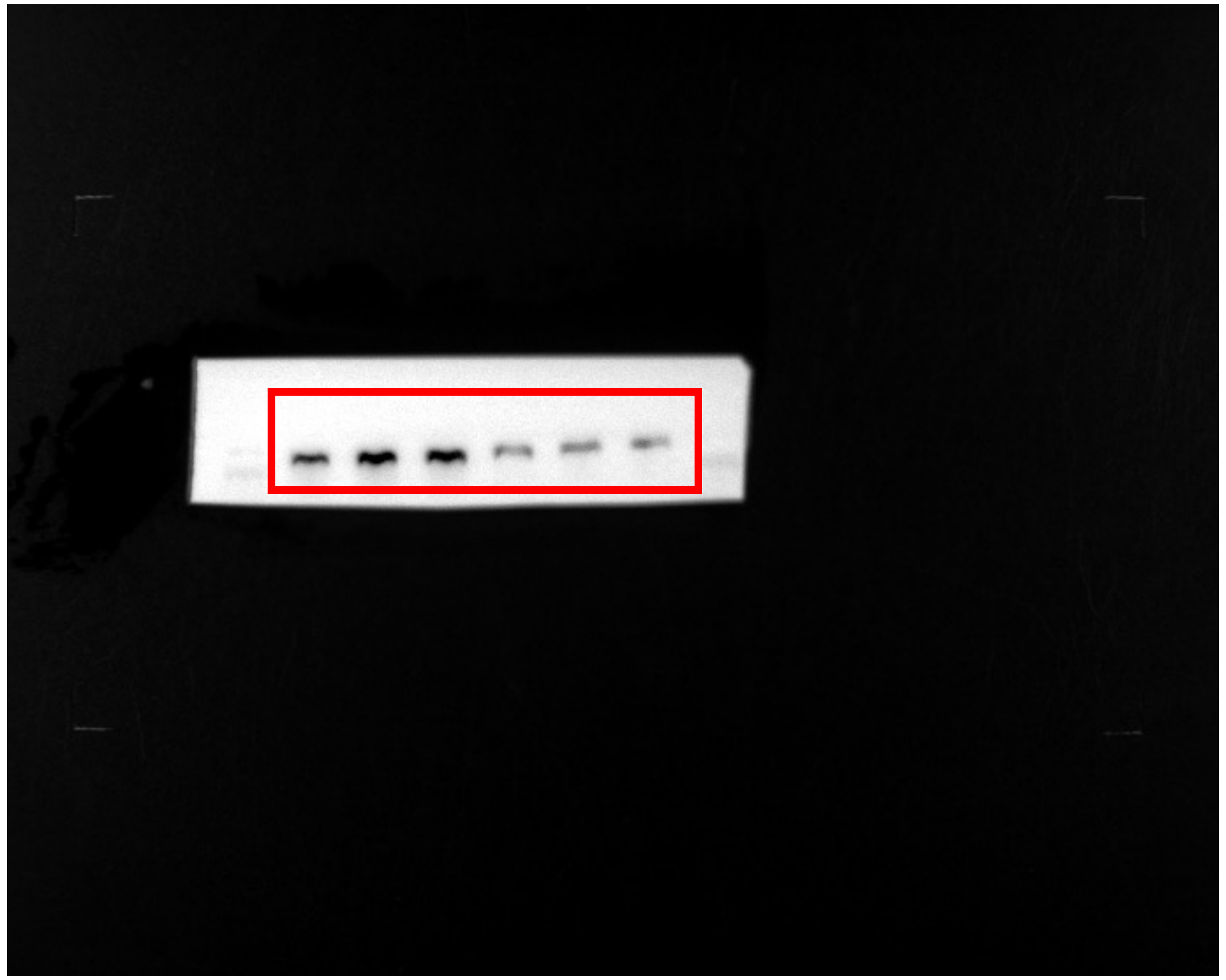

H3

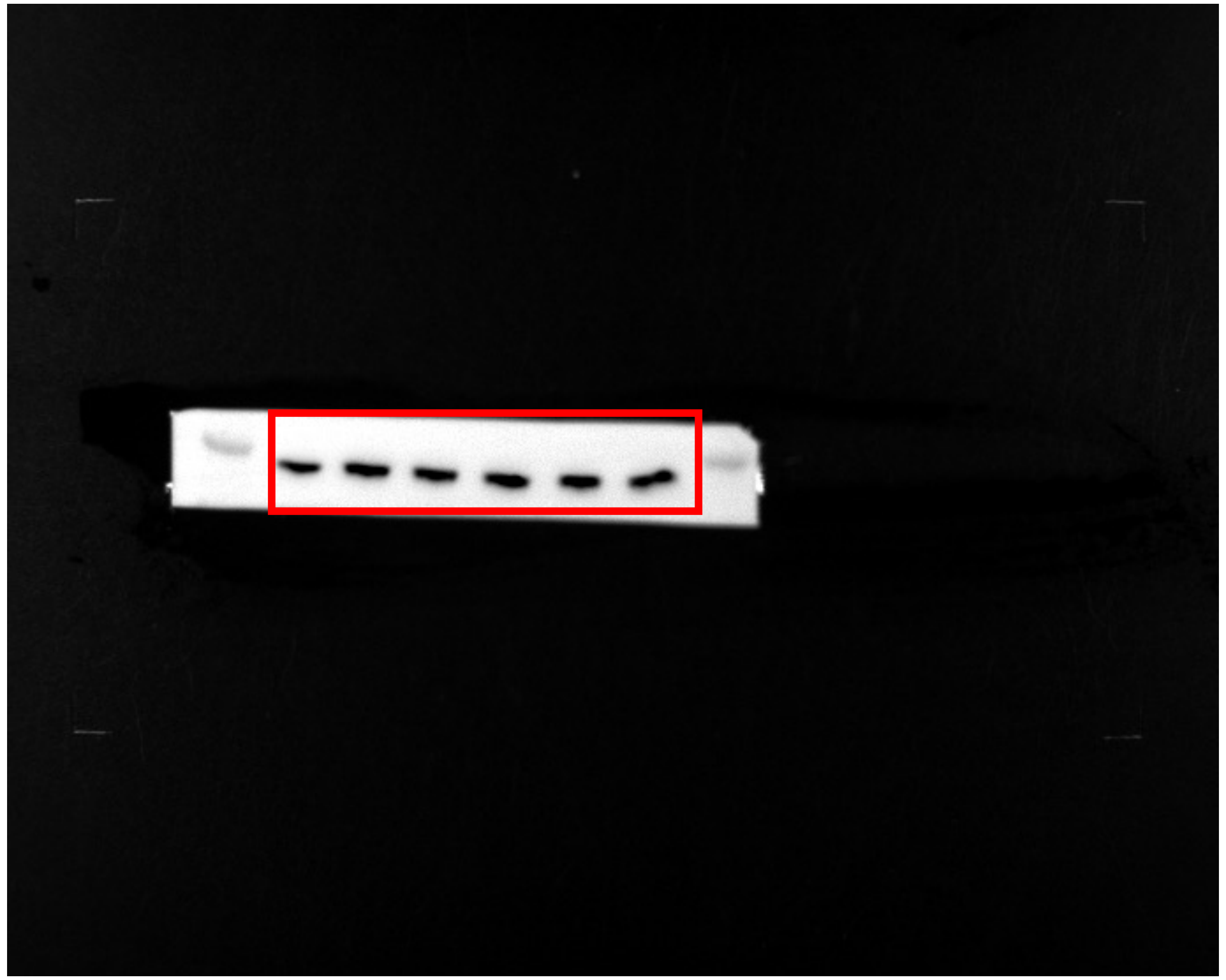

Full unedited blots for Figure S6D

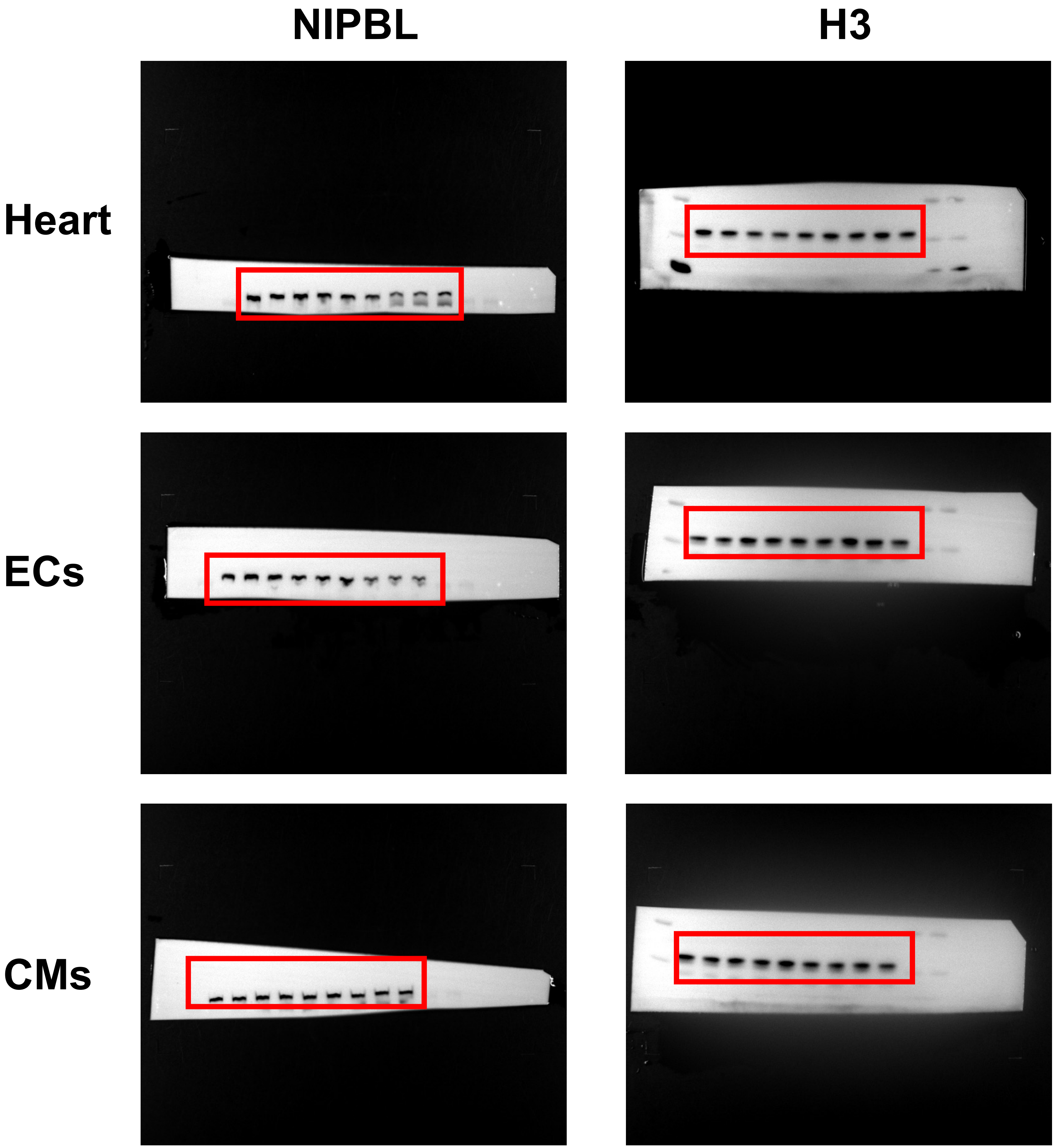

Full unedited blots for Figure S6E

NIPBL

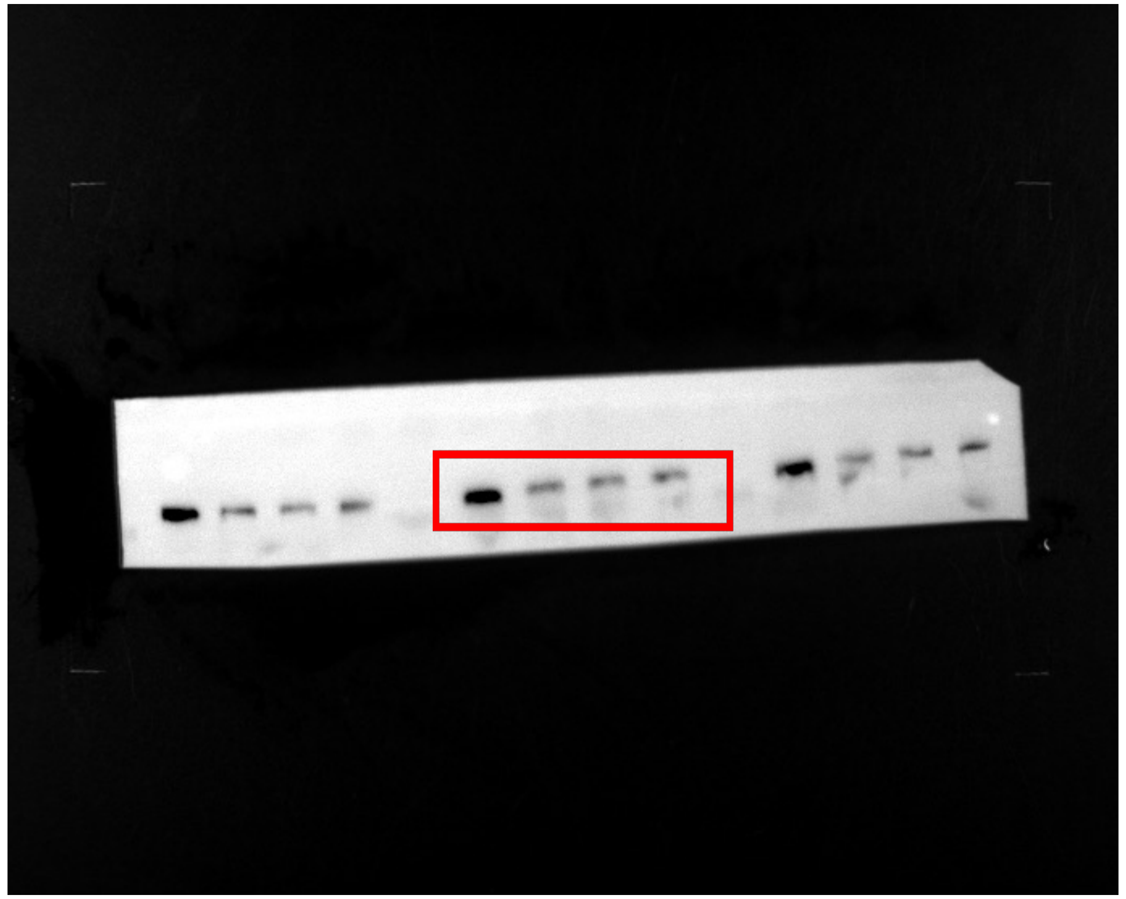

H3

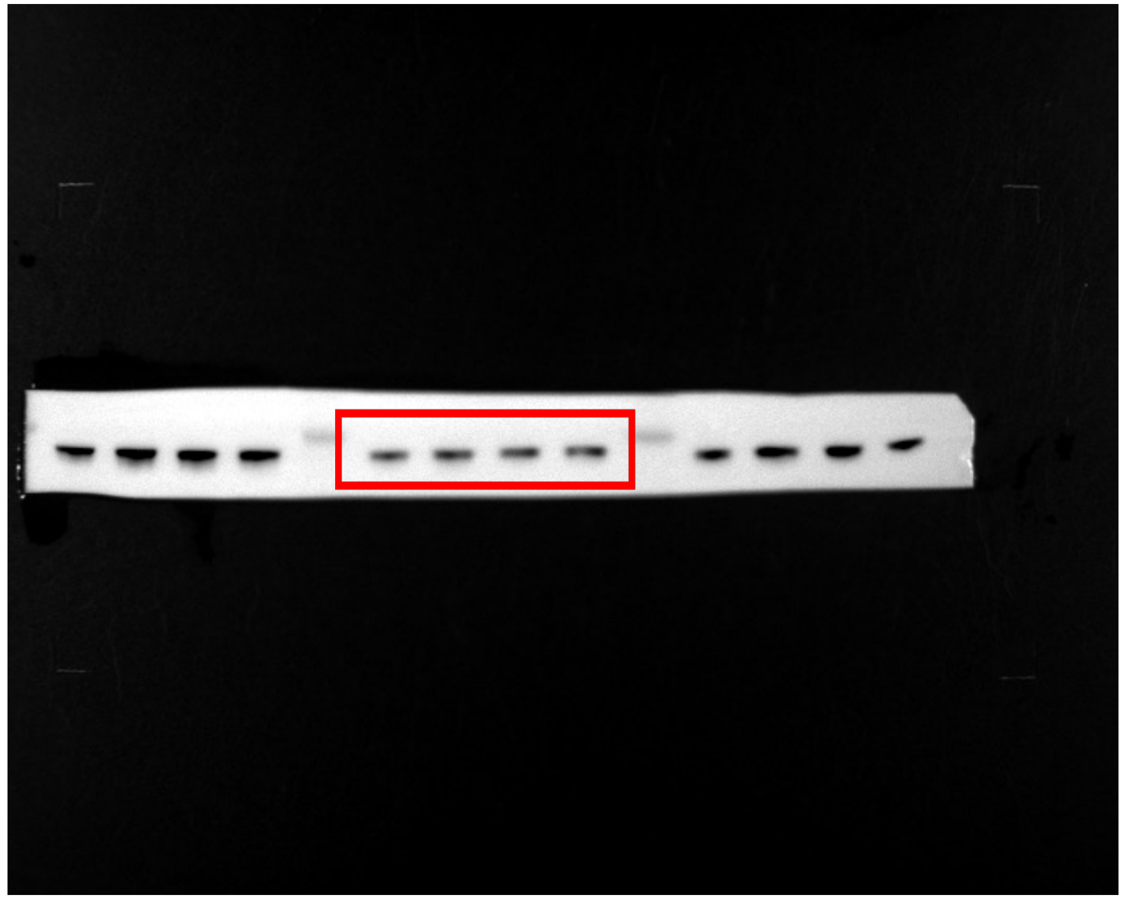

Full unedited blots for Figure S8B

NIPBL

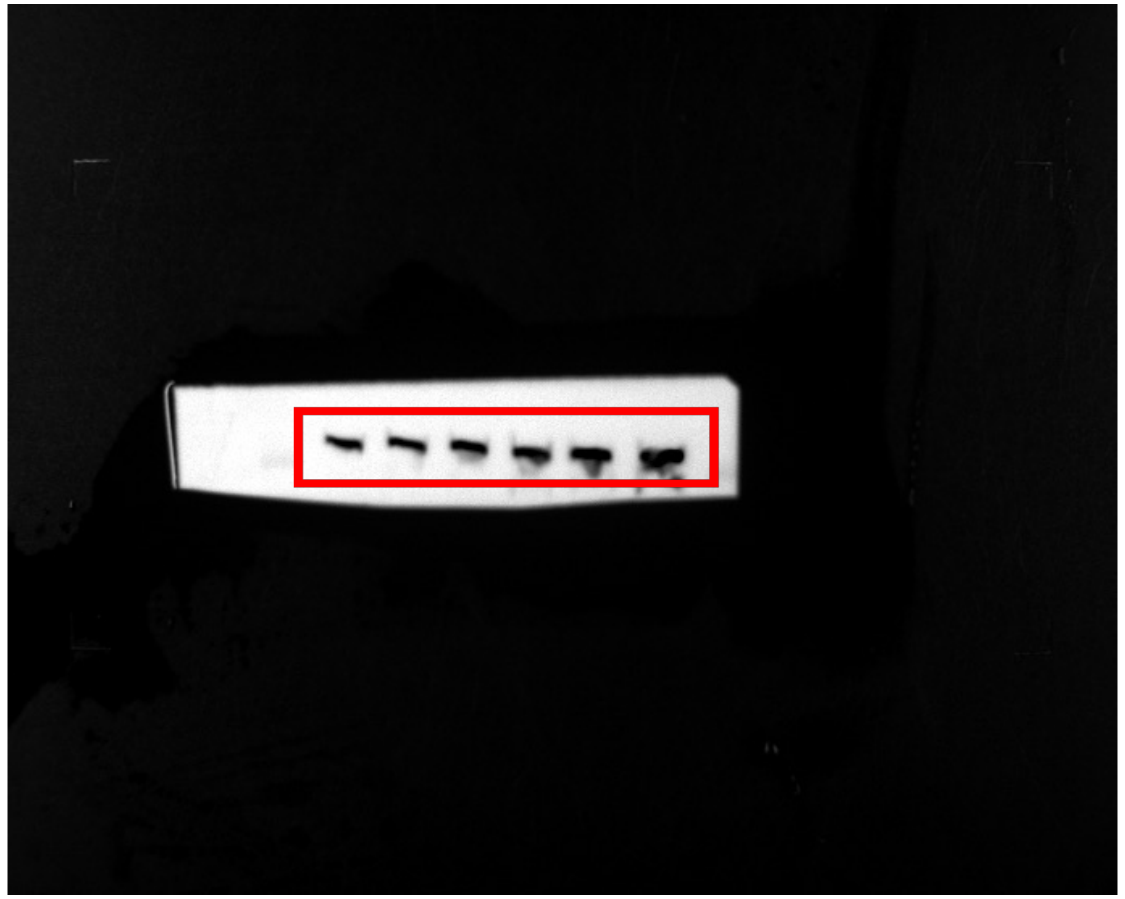

H3

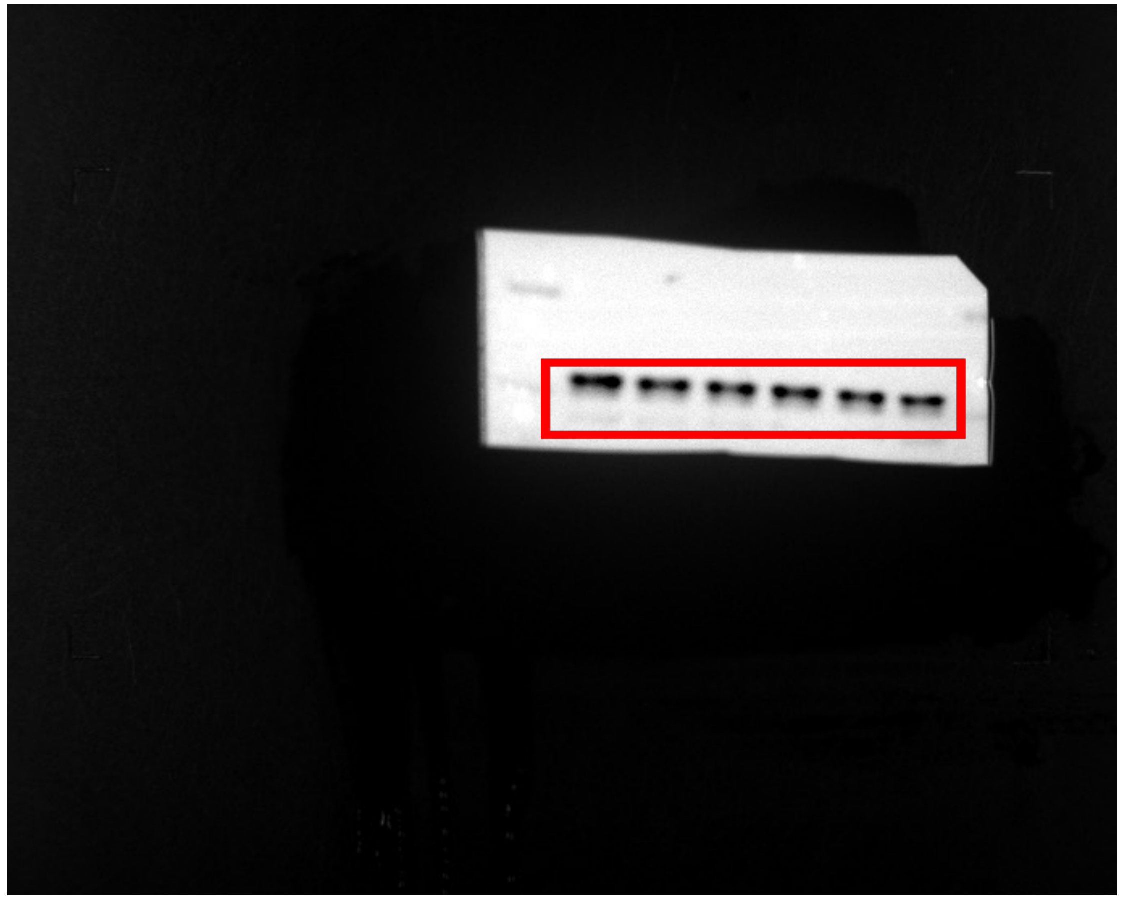

Full unedited blots for Figure S10B

N-cadherin

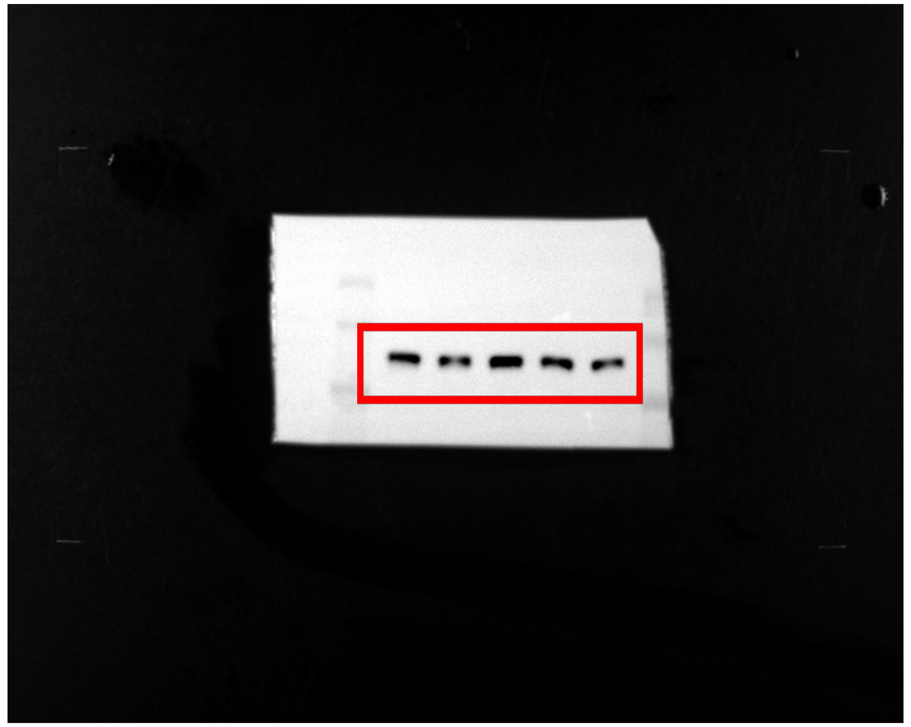

E-cadherin

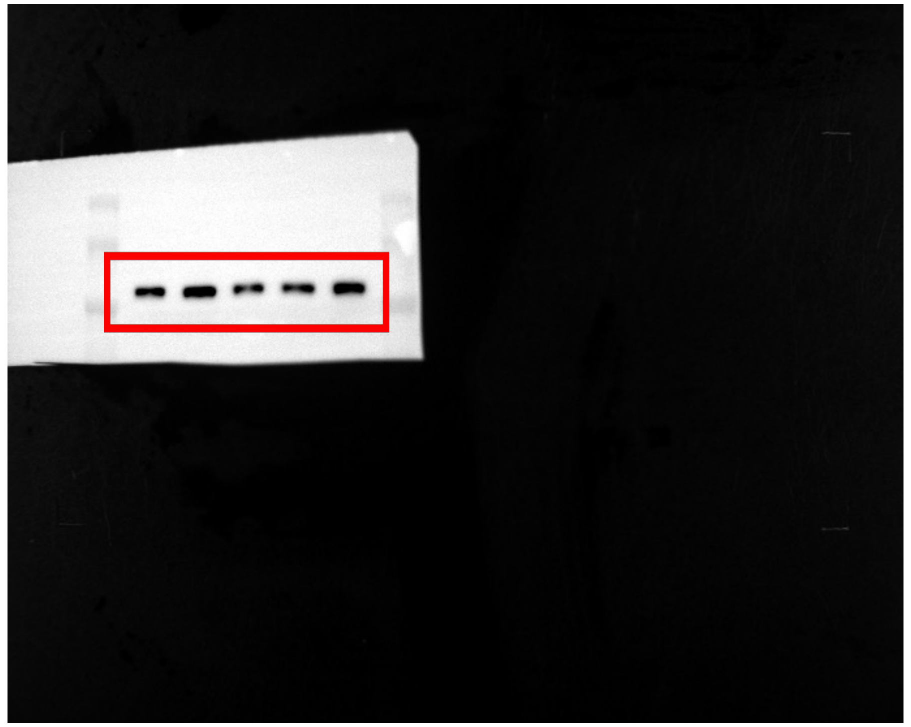

GAPDH

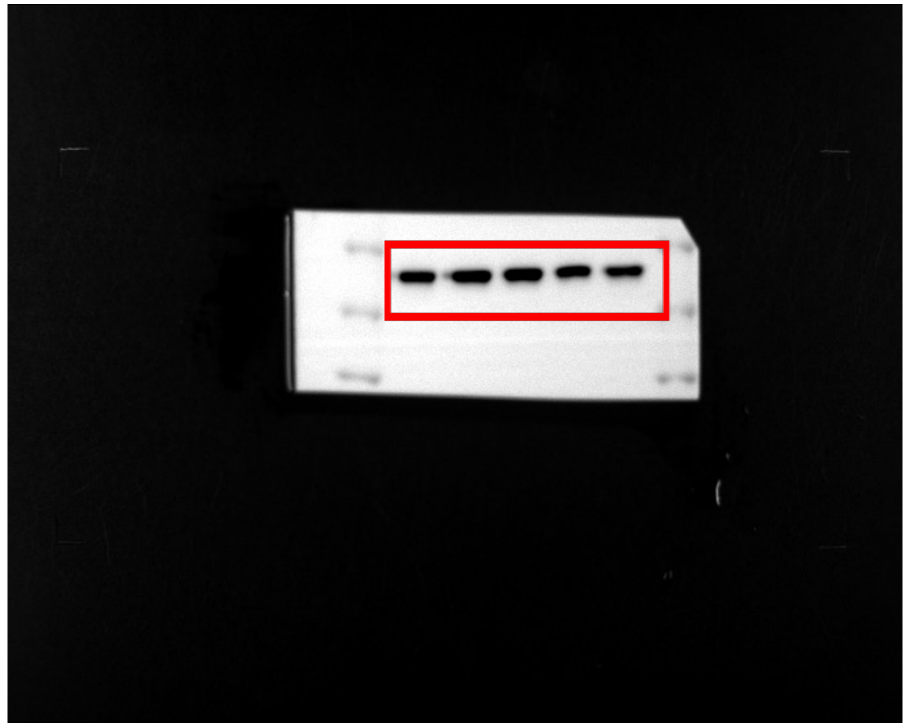

Supplement: Unedited blot and gel images [file jci-135-178355-s116.pdf]
